# Chronic obstructive pulmonary disease (COPD) and autoimmune diseases: unravelling complex interactions

**DOI:** 10.1080/07853890.2025.2587931

**Published:** 2025-11-19

**Authors:** Alexandr Ceasovschih, Victorița Șorodoc, Anastasia Balta, Mihai Constantin, Bianca Codrina Morărașu, Alexandra-Diana Diaconu, Raluca-Elena Alexa, Andreea Asaftei, Mariana Pavel-Tanasa, Gabriela Rusu-Zota, Alexandru Corlăteanu, Georgia Hardavella, Laurențiu Șorodoc

**Affiliations:** ^a^ Grigore T. Popa University of Medicine and Pharmacy, Iasi, Romania; ^b^ Department of Pulmonology and Allergology, “Nicolae Testemitanu” State University of Medicine and Pharmacy, Chisinau, Moldova; ^c^ 6^th^ Department of Respiratory Medicine, “Sotiria” Athens’ Chest Diseases Hospital, Athens, Greece

**Keywords:** Chronic obstructive pulmonary disease, COPD, autoimmunity, autoimmune diseases

## Abstract

**Background:**

Chronic obstructive pulmonary disease (COPD) is a global healthcare problem, characterized by a progressive and irreversible decline in lung function, primarily due to airflow limitation caused by inflammation and emphysema. While smoking is the main risk factor, systemic inflammation also plays a significant role in the development and progression of the disease, particularly in relation to autoimmune conditions.

**Research question:**

This literature review provides an overview of current evidence regarding the underlying pathophysiological mechanisms that link autoimmune inflammatory processes to COPD. It also examines the clinical relevance of these associations, with a focus on potential diagnostic and therapeutic implications.

**Methods:**

To ensure a comprehensive perspective, a broad literature search was conducted in PubMed and Embase in March 2025, using a wide range of search terms related to COPD and multiple autoimmune conditions. Relevant studies of any design were included if they provided valuable insights into the interplay between autoimmune processes and COPD, while non-English publications and commentaries were excluded.

**Results:**

Studies suggest a potential association between COPD and autoimmune processes, with chronic systemic inflammation playing a central role. The evidence points to immune dysregulation as a contributing factor to both COPD progression and its connection to autoimmune conditions.

**Conclusion:**

The complex interactions between COPD and autoimmune diseases require further investigation. Gaining a better understanding of these interactions may provide new insights into managing patients with concurrent pulmonary and autoimmune conditions, emphasizing a growing area of clinical research.

## 1. Introduction

Chronic obstructive pulmonary disease (COPD) is a global healthcare problem, with an estimation of approximately 600 million cases by 2050 [1].

COPD is characterized by an irreversible decline in lung function mainly due to airflow limitation caused by a wide variety of multiple risk factors, with smoking being the most significant among all [2–5]. COPD may affect both small and larger airways, as well as lung parenchyma where inflammation and autoimmunity play a key role in COPD progression and evolution [6,7].

The innate and adaptive immune systems are the primary mechanisms of body defense, responsible for the pathophysiologic reactions in COPD [8]. It is widely known that COPD and autoimmune conditions, such as rheumatoid arthritis (RA) and systemic lupus erythematous (SLE), share smoking as a common risk factor. In addition, several other pathophysiological mechanisms may be implicated. The shared embryonic mesodermal origin of the lungs and other organs may predispose them to similar injury pathways. Oxidative stress, through processes such as protein carbonylation, citrullination and the degradation of elastin, can result in the formation of molecular fragments that act as neoantigens, triggering immune responses. These antigens can subsequently drive the activation of both the innate and adaptive immune systems, leading to chronic inflammation and autoimmune reactions that contribute to progressive lung tissue destruction and emphysema development [9,10].

The aim of this review is to unravel the complex relationship between systemic autoimmune diseases and COPD development. Various autoimmune diseases are known to induce pulmonary manifestations, most frequently resulting in a restrictive spirometry pattern due to inflammation and fibrotic changes within the lung parenchyma [11,12]. However, there is growing interest in understanding the potential link between these diseases and obstructive respiratory impairment. We aim to describe the pathophysiological mechanisms that may link autoimmune processes with COPD, as well as examine the clinical implications of these findings. By focusing on the overlap between autoimmune diseases and COPD, this review aims to highlight an emerging area of research that could provide new insights into patient management and treatment with these concurrent conditions.

A comprehensive literature research was conducted in March 2025 in PubMed and Embase databases. The main search items included ‘chronic obstructive pulmonary disease’, ‘COPD’, ‘autoimmunity’, ‘autoimmune diseases’, ‘rheumatoid arthritis’, ‘systemic lupus erythematosus’, ‘systemic sclerosis’, ‘axial spondyloarthritis’, ‘Sjögren syndrome’, ‘psoriasis’, ‘psoriatic arthritis’, ‘antiphospholipid antibody syndrome’, ‘polymyalgia rheumatica’, ‘inflammatory bowel diseases’, ‘multiple sclerosis’, ‘Guillain-Barré syndrome’, ‘pernicious anemia’, ‘type 1 diabetes mellitus’, ‘thyroid’ and ‘thyroid dysfunction’. These terms were combined using the logical operators ‘OR’ and ‘AND’. We included any relevant publications that explored the connection between COPD and autoimmune inflammatory processes, regardless of study design, as long as they provided valuable insights into the topic. Exclusion criteria included commentaries and non-English language articles.

The findings from this broad body of literature were critically reviewed and synthesized into a narrative overview. The discussion first addresses the mechanisms linking COPD to autoimmune diseases, then provides an overview of the evidence available for specific autoimmune conditions, and finally considers the potential clinical implications. Where appropriate, relevant clinical studies are summarized in tables to facilitate comparison and interpretation.

## 2. Mechanisms linking COPD and autoimmune diseases

In COPD pathophysiology, both adaptive and innate immune systems possess significant roles, with inflammation being caused predominantly by innate immunity [13].

Innate immunity is comprised of physical barriers (airway epithelial cells) and specialized cells (dendritic cells, macrophages, neutrophils, monocytes, eosinophils), while adaptive immunity involves B and T cells as well as bronchus-associated lymphoid tissue (BALT). Both types of immunity are responsible for the proinflammatory cytokines’ secretion [8,14].

The adaptive immune system is activated by environmental stimuli such as cigarette smoke (CS), pollutants, infectious agents, self-antigens, the imbalance between protease and antiprotease levels and gene expression dysregulation [3].

Self-antigens arise from extracellular matrix degradation, post-translational protein modifications (protein carbonylation and citrullination), or cellular debris. These self-antigens can induce an adaptive immune response, activating numerous immune pathways that trigger and propagate the immune response and chronic inflammation, ultimately leading to the parenchymal destruction and emphysema development [15].

In COPD patients, elastin fibers are the main extracellular matrix component responsible for disease development. Activated neutrophils and macrophages release proteolytic enzymes damaging the pulmonary tissue and leading to emphysema [9].

A key component in the lung tissue damage seen in COPD is the destruction of elastin fibers in the extracellular matrix. Elastin fragments are released after elastic fiber degradation by elastase and matrix metalloproteinases, inducing the formation of interferon gamma and elastin antibodies. In a study conducted by Zhou et al. [10], mice exposed to CS and high elastin levels had a Th-1 immune response, with elastin fragments acting as self-antigens, activating immune memory.

Oxidative stress plays a pivotal role as well in the initiation and disease progression, the most important mechanism being protein carbonylation, which induces irreversible oxidation of amino acids (lysine, serine, arginine, proline, threonine). Protein carbonylation subsequently leads to antigen formation and adaptive immunity activation, promoting a Th1-type response [16]. The presence of IgG1 antibodies against carbonyl-modified proteins has been proposed as a potential trigger for the disease [17].

Another mechanism responsible for protein modification is citrullination, a process induced by hypoxia in COPD patients. Even though serum citrullinated proteins are associated with RA, high serum levels may also be found in COPD, indicating the inflammatory state of the disease, linking COPD with RA. In a study by Lugli et al. [18] it was shown that lung inflammation in COPD can promote the production of anti-citrullinated protein antibodies (ACPA).

Additionally, when cells undergo apoptosis, intracellular antigens can be exposed to the surface of dying cells, which may be recognized as self-antigens. Intracellular antigens, once released onto the surface of the apoptotic cells, are recognized as self-antigens and autoantibodies are produced. Finally, the binding of autoantibodies to self-antigens on the surface of apoptotic cells leads to immune tolerance and autoimmunity [7].

**Figure 1.**
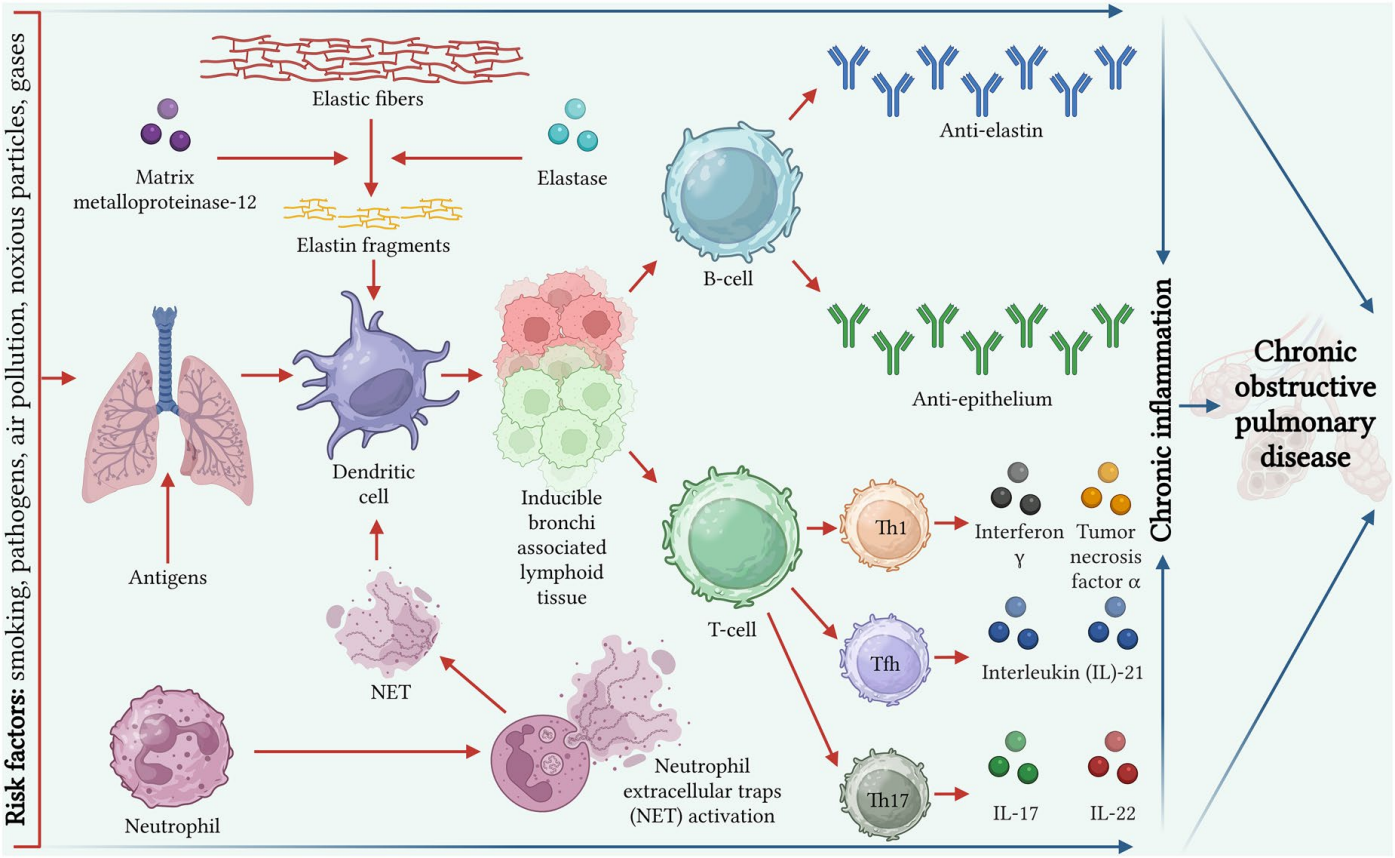
Pathophysiological mechanisms linking COPD and autoimmunity. Risk factors such as cigarette smoke, air pollutants and pathogens induce epithelial cell injury, oxidative stress and the activation of inflammatory cells, including alveolar macrophages and neutrophils. Activated neutrophils and macrophages release proteolytic enzymes, such as matrix metalloproteinase-12 and elastase, which degrade elastic fibers in the lung extracellular matrix. This process generates elastin fragments and carbonylated proteins that act as self-antigens. Neutrophil extracellular traps further contribute to antigen exposure. These self-antigens are processed by dendritic cells, leading to the development of inducible bronchi associated lymphoid tissue and the activation of B and T lymphocytes. B-cells differentiate into plasma cells producing autoantibodies against elastin and epithelial components. Activated T-helper 1 (Th1), T-helper 17 (Th17) and T follicular helper (Tfh) cells secrete proinflammatory cytokines, including interferon-γ (IFN-γ), tumor necrosis factor-α (TNF-α), interleukin-17 (IL-17), interleukin-21 (IL-21) and interleukin-22 (IL-22). This cascade perpetuates chronic airway inflammation and parenchymal tissue destruction in COPD. (Created with BioRender.com).

Overall, these mechanisms highlight how innate and adaptive immune responses, oxidative stress, and self-antigen generation collectively sustain chronic inflammation and autoimmunity in COPD. These interconnected pathways are illustrated in Figure 1, which summarizes the main steps linking extracellular matrix degradation, autoantibody production, and chronic lung tissue damage.

### 2.1. Innate immune system

There are several mechanisms involved in the activation of the innate immune system in COPD:

#### 2.1.1. Secretion of pro-inflammatory mediators

The stimulation of the lung’s epithelial cells triggers the secretion of pro-inflammatory mediators, which activate the innate immune system and the nuclear factor kappa-light-chain-enhancer of activated B-cells (NF-κB) signaling pathway. Following this activation, there is an upregulation of the genes responsible for the secretion of inflammatory mediators (IL-1A, IL-1B, IL-6, C-C motif chemokine ligand 20, colony-stimulating factor-1, NF-κB inhibitor 1-α, NFκB inhibitor 1-ζ, and downregulation of the genes responsible for anti-inflammatory mediators’ secretion (such as fatty acid binding protein 5) [9,14,19].

The activation of NF-κB pathway in COPD stimulates the transcription of inflammatory cytokine genes and leads to increased concentrations of pro-inflammatory cytokines (IL-8, IL-1β, GM-CSF, TNF-α, IFN-β). These cytokines drive and maintain chronic inflammation by recruiting and activating elements of the innate immune system. Cytokines from NF-κB pathway and INF-β play an important role in autoimmune diseases [14].

#### 2.1.2. Neutrophil dysfunction

Neutrophils, integral components of the innate immune response, exhibit a critical role in the inflammatory processes associated with COPD [3]. Their main functions involve degranulation, phagocytosis of viruses or antigens and neutrophil extracellular traps (NETs) release [9]. However, in COPD patients, neutrophils release a large number of NETs and reactive oxygen species (ROS), but exhibit decreased phagocytosis [3]. In patients with COPD, there is a dysfunction in neutrophil activity, possibly due to decreased chemoattractant response [2]. After activation, neutrophils release ROS, elastase and inflammatory cytokines that promote and maintain inflammation and emphysema [20]. Elastase, produced by neutrophils, independently leads to emphysema [2].

Another mechanism involved is neutrophil extracellular trap (NETosis), a process in which neutrophils release antibacterial proteins and neutrophilic chromatin from granules entrapping different antigens. These are web-like structures regulated by C-X-C motif chemokine receptor 2, which has been correlated with COPD severity [21].

#### 2.1.3. Matrix-metalloproteinase (MMP) secretion

Alveolar macrophages further contribute to lung inflammation through structural damage of lung parenchyma by MMPs. These macrophages release a variety of products, including proteases, cytokines, and chemokines, which promote the expression of MMPs and facilitate structural damage to lung parenchyma. In fact, alveolar macrophages may cause long term immune deficiency of lung tissue after exposure to different antigens (viral, bacterial, self-antigens, etc.) [9].

Beyond classical pathogen recognition, innate immunity in COPD can sustain chronic inflammation through the persistent release of damage-associated molecular patterns (DAMPs), such as mitochondrial components and extracellular matrix fragments. These endogenous DAMPs engage pattern recognition receptors (PRRs) on dendritic cells and macrophages, amplifying inflammatory signaling cascades even in the absence of infectious triggers [22]. Importantly, this continuous DAMP-driven activation may break peripheral tolerance, enhancing autoantigen presentation and promoting the emergence of autoreactive T and B cell clones, thus linking innate immune dysregulation to the development of autoimmune features in COPD [22,23]. Recent evidence highlights that classical and novel DAMPs – including HMGB1 and mitochondrial DNA – can activate PRRs such as TLRs and RAGE, reinforcing NF-κB signaling and inflammasome pathways. This sustained innate stimulation further erodes self-tolerance and is increasingly recognized as a bridge to autoimmunity in chronic inflammatory diseases, including COPD [23].

### 2.2. Adaptive immune system

There are several mechanisms leading to the activation of the adaptive immune system in COPD, involving both cellular and molecular responses:

#### 2.2.1. Dendritic cells (DCs)

Upon activation, DCs upregulate molecules such as C-X-C motif receptor **7** and major histocompatibility complex (MHC) class I and II, further activating CD4+ T cells. This activation contributes to the formation of BALT, composed of B-cells, T-cells, and conventional DC type 2 (cDC2) [9]. The migratory signature of cDC2 and their role in promoting IL-21 secretion *via* the OX40-OX40L axis make them crucial in BALT formation and functionality in COPD [24].

Moreover, by capturing self-antigens from damaged lung structures and upregulating costimulatory pathways, DCs maintain a local microenvironment that supports the expansion of autoreactive T helper and T follicular helper cells, promoting sustained B-cell activation and immunoglobulin switching within BALT, and thereby reinforcing the autoimmune features increasingly recognized in COPD [9,25].

#### 2.2.2. B-cells

In COPD patients, lymphoid nodules rich in B-cells and lymphatic microvascular remodelling correlate with disease severity [26,27]. Plasma cells derived from B-cells produce autoantibodies, predominantly IgG, compared to the IgA dominance in healthy individuals. The formation of autoantibodies in COPD leads to lung tissue destruction through complement system activation [3,9]. Moreover, B-cells also present antigens to T-cells *via* MHC class I and II [26].

Experimental models demonstrate that B-cell-deficient mice are resistant to cigarette smoke-induced emphysema, further supporting the pathogenic role of B-cells in lung injury [28,29]. B-cells also engage in local antigen presentation and class switching within BALT structures, which fosters chronic autoreactivity and perpetuates local immune activation. Additionally, increased expression of B-cell activating factor (BAFF) and C-X-C Motif Chemokine Ligand 13 (CXCL13) within COPD lung tissue has been shown to sustain B-cell survival and follicle formation, reinforcing the autoimmunity loop [9,30].

#### 2.2.3. T-cells

Both CD4+ and CD8+ T-cells have shown significant associations with COPD severity [9,26]. CD4+ T-cells differentiate into Th-17 cells, which secrete IL-17, contributing to emphysema through neutrophil activation and perpetuating chronic inflammation. Regulatory T-cells (Tregs) help control autoimmunity and are divided into resting Tregs, active Tregs, and cytokine-secreting pro-inflammatory Tregs. In COPD patients, the anti-inflammatory Tregs are reduced compared to the pro-inflammatory counterparts [3,19]. Among immune cells, only Tregs show a negative correlation with COPD severity [3]. The sustained expansion of Th1, Th17 and CD8+ T-cells, combined with the shift towards pro-inflammatory Treg subsets, supports chronic autoreactive responses by lowering peripheral tolerance and perpetuating local self-antigen presentation, which may directly contribute to the autoimmune features of COPD [9].

#### 2.2.4. CD8+ T cells and Th1 cells

Stimulation of CD8+ T-cells and Th1 cells with IL-18 and IL-12 increases the production of IFN-γ and TNF-α, while IL-15 promotes the release of perforin, leading to alveolar cell apoptosis, emphysema, and the sustenance of immune responses through antigen release [31].

In patients with COPD, a dysregulation of the immune response may occur, influenced by chronic exposure to environmental factors, such as CS and infections which lead to persistent inflammation and lung tissue damage [32]. This dysregulation affects Tregs, reducing their ability to suppress excessive immune responses. Inadequate activation of B and T cells within the BALT leads to complement pathway activation *via* C3a and the autoantibodies formation [9]. BALT is formed mainly from B-cells, which are activated by the B-cell activating factor, a cytokine in the TNF family implicated in B-cell homeostasis, activation and differentiation of monocytes to macrophages, DCs stimulation, and immune cell accumulation at the inflammation site [13].

CD57+ cells represent a terminally differentiated subset of cytotoxic lymphocytes, primarily arising from CD8+ T cells and Natural Killer-like cells that acquire high cytolytic potential and features of immunosenescence. Within the lung microenvironment of COPD patients, Olloquequi et al. [33] demonstrated that the density of CD57+ cells is significantly increased in pulmonary lymphoid follicles compared to healthy controls. This accumulation suggests a sustained local cytotoxic response and immune dysregulation, promoting chronic inflammation and structural tissue damage through persistent activation of tertiary lymphoid structures. Interestingly, similar expansions of CD57+ T cell subsets have been documented in various autoimmune diseases, including rheumatoid arthritis, systemic lupus erythematosus, spondyloarthritis and type 1 diabetes, where they are linked to immunosenescence, sustained cytotoxicity, and aberrant inflammatory responses [34–37]. This convergence further supports the hypothesis that CD57+ cells may represent a shared immunopathological mechanism bridging COPD with systemic autoimmunity.

## 3. COPD and rheumatic diseases

Rheumatic diseases are conditions in which the immune system mistakenly attacks the body’s own tissues, causing inflammation, pain, and damage to joints, muscles, and other organs. These diseases are chronic and can have systemic effects, impacting on multiple organs and leading to significant morbidity and disability [38].

COPD and rheumatic diseases are chronic processes which significantly affect the quality of life. Both diseases share common risk factors and inflammatory pathways, despite different organ systems being affected, leading to a high prevalence of rheumatic diseases among COPD patients ranging up to 50%. Smoking, aging, and genetic predisposition are the main risk factors implicated in both conditions, with smoking being the most prevalent [39]. Understanding the interplay between these conditions is crucial for comprehensive patient care and it subsequently necessitates a multidisciplinary approach to diagnosis, management, and treatment [38].

Additionally, systemic inflammation is a hallmark of both conditions, suggesting potential pathophysiological links. Chronic inflammation is central to both COPD and rheumatic diseases. Pro-inflammatory cytokines, such as TNF-α and IL-6, are elevated in both conditions. This common inflammatory milieu may explain the higher prevalence of COPD among patients with rheumatic diseases and vice versa [40–42].

Rheumatic diseases frequently involve the lungs, presenting as interstitial lung disease (ILD), pleuritis, and pulmonary hypertension (PH). These pulmonary manifestations may complicate the clinical course of pre-existing COPD as well as its diagnosis and management [43].

### 3.1. COPD and rheumatoid arthritis

RA is a systemic autoimmune disease, most commonly characterized by symmetrical inflammation of the small peripheral synovial joints. Over time, patients may develop extra-articular ocular, oral, pulmonary, cardiac, gastrointestinal, renal, hematological and dermatological manifestations [43,44]. RA can be subdivided into seropositive and seronegative depending on the presence of ACPA in serum. Serum positivity and negativity relate to subsequent differences in the etiology pathophysiology, clinical phenotypes, and treatment response of patients [45].

Recent research has shown that patients with seropositive RA face a significantly increased risk of developing COPD. Specifically, Rossides [46] reports that ACPA-positive patients have a 26% higher risk of COPD than ACPA-negative patients. This association is attributed to smoking’s ability to exacerbate inflammation, induce epigenetic changes and cause the production of ACPA, which then may cause RA.

In addition to the established role of smoking-induced ACPA production, emerging evidence highlights local lung citrullination as a potential initiator of systemic autoimmunity. NETs and peptidylarginine deiminase (PAD) enzymes in inflamed COPD airways promote the generation of citrullinated neoantigens that may sustain or amplify ACPA positivity [9,47]. Furthermore, the presence of the HLA-DRB1 shared epitope provides additional genetic susceptibility by enhancing antigen presentation and autoantibody production [48,49].

However, emerging evidence suggests that the relationship between RA and COPD is more complex than previously understood, suggesting the presence of other factors contributing to this bivalent relationship. RA has been shown to lead to pulmonary flora imbalance and an overpopulation of *Pseudomonas.* This dysbiosis leads to IL-17 production, triggering the production of cytokines IL-1β, IL-6, and IL-23, and subsequently contributing to COPD pathogenesis [50,51]. ACPAs present in the synovial joints have been implicated in the increased prevalence of COPD in this group of patients. These antibodies are believed to contribute to COPD through their role in the systemic inflammatory processes and immune dysregulation associated with RA, thereby linking RA to a higher incidence of COPD. Additionally, chronic and persistent inflammation owing to RA may lead to destruction of the airways and facilitate COPD formation [50]. COPD patients often produce elevated levels of self-antigens, which could contribute to the development of RA, highlighting a bidirectional relationship [52]. Finally, Hemminki et al. [53] investigated the association between RA, COPD, and lung cancer. Their results indicated that RA patients are more prone to COPD development than in lung cancer, suggesting that autoimmunity may contribute to the increased risk of COPD (Table 1). Despite established links RA and COPD, the underlying mechanisms remain complex and multifaceted. Further research is essential to unravel these intricate interactions and to improve strategies for the prevention, diagnosis, and management of both RA and COPD.

**Table 1.** Characteristics of selected studies on COPD and RA.

Reference	Study Design	Patients	Outcomes/conclusions
Sparks et al. [54] 2018	Prospective cohort study	843 females diagnosed with RA and 8,399 healthy cohorts	- COPD incidence: 8,1% in women with RA, 5,5% in cohorts. - RA significantly increased COPD risk (HR 1.68). - Both seropositive and seronegative RA had a similar increased risk of COPD.
Nannini et al. [55] 2013	Retrospective cohort study	594 RA patients and 596 healthy cohorts	− 52 RA patients and 40 healthy cohorts developed COPD. - RA patients had a significantly higher risk of COPD (HR: 1,54).
Dougados et al. [56] 2018	International, cross-sectional study	3,920 RA patients from 17 countries	− 3,5% of RA patients had COPD.
Bieber et al. [57] 2013	Cross-sectional analysis	9,039 RA patients and 15,070 controls	- COPD was more common in RA patients (8,6%) compared to controls (4,4%). - RA patients had more than twice the odds of having COPD compared to controls.
Shen et al. [58] 2014	Retrospective cohort study	28,725 newly diagnosed RA patients and 114,900 controls	- RA patients had a 1,74-fold higher incidence of COPD.
Ursum et al. [59] 2013	Nested case-control study	3,354 inflammatory arthritis patients and 6,708 controls.	- RA patients had a 1,8 times higher risk of developing COPD.
McGuire et al. [60] 2017	Retrospective cohort study	24,625 RA patients and 25,396 healthy cohorts	- RA patients had a 47% higher risk of COPD hospitalization.

COPD: Chronic obstructive pulmonary disease; HR: Hazard ratio; RA: Rheumatoid arthritis.

### 3.2. COPD and systemic lupus erythematosus (SLE)

SLE is a multisystem autoimmune disorder with a predilection for young females. Within the Caucasian population, SLE has an incidence rate of approximately 0.67%, whereas in the African American population, the incidence rate is significantly higher at approximately 3.14% [61]. The clinical presentation of SLE is heterogeneous, with potential involvement of nearly all organ systems, including, but not limited to, neurological, psychiatric, ocular, dermatological, musculoskeletal, cardiovascular, pulmonary, renal, gastrointestinal, endocrinological, obstetrical, and hematological systems. Systemic complications are also quite prevalent, with cardiovascular disease affecting up to 6.5% of patients, neurological complications ranging from 3% to 20%, and respiratory involvement in approximately 50% of patients [62–64].

The immunopathogenesis of SLE is characterized by the production of a variety of autoantibodies, most notably anti-nuclear antibodies (ANA). Other specific autoantibodies commonly observed in SLE patients include anti-Scl-70, anti-La, anti-Ro, anticardiolipin, and anti-phospholipid antibodies. The presence of these diverse autoantibodies is not only a hallmark of SLE but it also suggests a strong correlation with other autoimmune disorders, indicating a shared pathogenic mechanism [61].

Lung involvement in SLE is multi-faceted and well-documented. Common pulmonary involvement includes pleuritis, acute lupus pneumonitis, diffuse alveolar hemorrhage, SLE-associated ILD, pulmonary venous thromboembolism, pulmonary arterial hypertension, and lung infections.

Recent studies support the correlation between SLE and COPD [65,66]. Although the exact mechanisms remain unclear, some studies suggest that as COPD progresses, the production of autoantibodies may increase, potentially predisposing patients to the development of SLE (Table 2) [72]. Several mechanistic pathways may underlie this association. Both SLE and COPD are characterized by excessive formation of NETs, which can expose autoantigens and perpetuate systemic autoimmunity. In addition, a type I interferon signature commonly found in SLE may be amplified in inflamed COPD airways, promoting B cell activation and autoantibody production. Smoking and oxidative stress further contribute to post-translational modifications of proteins, generating novel autoantigens that sustain chronic inflammation [68,71,73]. These overlapping mechanisms may explain how pulmonary inflammation in COPD could trigger or exacerbate autoimmunity in patients predisposed to SLE.

**Table 2.** Characteristics of selected studies on COPD and SLE.

Reference	Study Design	Patients	Outcomes/conclusions
Avina-Zubieta et al. [67] 2014	Retrospective matched cohort study	4,486 SLE patients and 44,860 controls	− 96 developed COPD, with an incidence rate of 4.96 per 1,000 person-years. - Relative risk of COPD in SLE patients was elevated (RR 2.31, 95% CI: 1.83–2.89) and remained elevated for up to 4 years.
Shen et al. [68] 2014	Retrospective cohort study	10,623 SLE patients and 42,492 controls	- COPD incidence was 1.73 times higher in SLE patients. Highest COPD risk was in the youngest SLE patients.
Katz et al. [69] 2021	Longitudinal cohort study	2,804 in one cohort, 881 in another cohort (both SLE patients)	− 8.3% reported COPD in the first cohort at baseline, 4.2% in the next 3 years. - In the second cohort, 35.6% reported asthma or COPD at baseline and 11.3% over the next 3 years.
Han et al. [70] 2017	Retrospective cohort study	1,680 emergency department visits due to SLE, 3,343 SLE hospitalizations	− 6.3% in males and 2.4% in females of emergency department visits in SLE patients were due to COPD. - COPD hospitalization rates were 6.9% in males over 60, 2.4% in females aged 40–59, and 3.7% in females over 60 years old with SLE.
Yu et al. [71] 2024	Mendelian randomization study	442 SLE patients and 218,254 cohorts	- Possible positive association, but MR Egger test does not show significant causal evidence (*p* = 0.405).

COPD: Chronic obstructive pulmonary disease; SLE: Systemic lupus erythematosus.

Continued research is imperative to elucidate these mechanisms and to evaluate the influence of COPD on SLE progression, in order to enhance diagnostic accuracy and optimize treatment strategies for patients affected by both conditions.

### 3.3. COPD and systemic sclerosis (scleroderma)

Systemic sclerosis (SSc), also known as scleroderma, is a rare autoimmune disorder affecting connective tissue. It is characterized by extensive fibrosis, vascular injury, and immune system dysfunction [74]. This disease is associated with high morbidity and mortality, often resulting from cardiopulmonary complications, such as ILD and PH [75].

The association between SSc and COPD is complex and involves multiple mechanisms and risk factors that may contribute to the development of both lung conditions.

COPD involves chronic airway inflammation, leading to airflow obstruction. Inflammation in SSc can exacerbate this process, potentially leading to an overlap syndrome where both obstructive and restrictive lung disease features are present [76]. Chronic inflammation and fibrotic changes in scleroderma can sometimes affect the airways, leading to symptoms similar to those of COPD. Smoking is a significant risk factor for COPD and can exacerbate pulmonary involvement in individuals with scleroderma, increasing the likelihood of respiratory complications. Also, both conditions can be influenced by environmental factors, such as exposure to pollutants, dust, and other irritants, which can exacerbate lung disease in susceptible individuals [77].

In a recent study conducted by Piplani et al. [78] researchers focused on patients with a principal diagnosis of COPD and a secondary diagnosis of scleroderma. The study aimed to explore COPD outcomes in individuals with scleroderma compared to the general population. This study found no significant correlation between COPD outcomes in patients with scleroderma and factors such as mortality, length of hospital stay, total charges, or the need for ventilation. However, descriptive statistics indicate minor differences between the groups, with a somewhat higher incidence of deaths and longer hospital stays noted in the scleroderma cohort.

A longitudinal retrospective study concluded that in patients with SSc and PH, the presence of COPD significantly increases the risk of all-cause mortality. Specifically, patients with both PH and COPD had a nearly three-fold higher risk of mortality compared to those with PH alone. This elevated risk was not observed in patients with PH and ILD or those with a combination of PH, ILD, and COPD. These findings suggest that COPD is a critical factor in worsening outcomes in this patient population, highlighting the need for early detection and management of COPD in patients with PH-SSc. Further research is needed to better understand the underlying reasons for this increased mortality risk and to confirm these results in larger cohorts [79].

Although the pathophysiology of scleroderma and associated pulmonary complications have been studied thoroughly, there are limited studies investigating the interaction and overlap of scleroderma and COPD (Table 3). A potential mechanistic link between the two conditions may involve autoimmune dysregulation in systemic sclerosis, including the production of autoantibodies and chronic immune cell infiltration, which could contribute to COPD-like airway damage. Shared pathological features, such as microvascular injury, persistent inflammation, and profibrotic cytokine release, may promote airway remodeling and airflow limitation [80]. The lack of specific data may also be due to the rarity of the simultaneous occurrence of these two conditions, as well as the difficulty in distinguishing between pulmonary complications caused by scleroderma and the typical symptoms of COPD. This can complicate diagnosis and treatment, as it can be challenging to differentiate between pulmonary symptoms arising from different causes that present with similar manifestations.

**Table 3.** Characteristics of selected studies on COPD and SSc.

Reference	Study design	Patients	Outcome/conclusions
Piplani et al. [78] 2024	Retrospective study	2,100 patients with scleroderma and 2,538,704 patients in the general COPD population	- No significant associations with mortality, length of stay, charges, or ventilation (*p* > 0.05). - Scleroderma group showed slightly higher death rates and longer stays.
Vakhshoorzadeh et al. [79] 2022	Retrospective study	162 patients with PH of SSc	- Patients with both PH and COPD had a higher hazard ratio for all-cause mortality than those with PH alone (*p* < 0.03).

COPD: Chronic obstructive pulmonary disease; PH: Pulmonary hypertension, SSc: Systemic sclerosis.

### 3.4. COPD and axial spondyloarthritis

Axial spondyloarthritis (axSpA) is a chronic inflammatory condition that can result in ankylosis due to the secondary ossification of inflammatory lesions. This process can lead to progressive disability and significantly affect the patient’s life quality. Pulmonary involvement is a recognized manifestation of axSpA, presenting either as ILD or as restrictive pulmonary function [81].

The association between axSpA and COPD is notable due to several shared factors. Patients with axSpA have a higher prevalence of COPD compared to the general population. This connection may be influenced by chronic inflammation, which is a common feature in both conditions, and shared risk factors, such as smoking. A possible mechanistic link involves the IL-17/IL-23 axis and overactivation of Th17 cells, central to axSpA pathogenesis and also implicated in airway inflammation. In addition, misfolding of HLA-B27 may promote chronic immune activation and cytokine release, potentially contributing to pulmonary damage [9,82]. Additionally, the COPD presence can exacerbate the overall disease burden in patients with axSpA, complicating treatment and management [83].

Our literature review included a study (Table 4) that examined data from the Swedish Hospital Discharge Register, comparing patients hospitalized for autoimmune diseases (from 1964 to 1999) with control subjects, specifically looking at the development of COPD in these groups. The analysis showed that patients with ankylosing spondylitis (AS) had a standardized incidence ratio of 1.72 for developing COPD, with a 95% confidence interval ranging from 1.23 to 2.33 [53].

**Table 4.** Characteristics of selected studies on COPD and axial spondyloarthritis.

Reference	Study Design	Patients	Outcome/conclusions
Hemminki et al. [53] 2011	Registry-based study	41 patients with AS	- Patients with AS had a standardized incidence ratio of 1.72 for developing COPD, with a 95% confidence interval ranging from 1.23 to 2.33.
Sharif et al. [83] 2018	Cross-sectional study	4076 patients with AS versus 20290 controls	- The odds ratio was 1.22, with statistical significance (*p* = 0.03), even after adjusting for age, gender, and smoking habits.
Zamout et al. [84] 2024	Case-control study	3571 patients with SpA were compared to 17855 matched controls	- The prevalence ratio was 1.03 (95% CI 0.85–1.24), indicating no significant difference.

AS: Ankylosing spondylitis; CI: Confidence interval; COPD: Chronic obstructive pulmonary disease; SpA: Spondyloarthritis.

A study from Israel, which included 4,076 patients with AS and an equal number of controls, found that AS was independently associated with an increased risk of developing COPD. The odds ratio was 1.22, with statistical significance (*p* = 0.03), even after adjusting for age, gender, and smoking habits [83].

Other studies have indicated that there is no significant association between COPD and spondyloarthritis (SpA). A recent case-control study found that the prevalence of COPD was not higher in patients with SpA compared to the general population. Analyzing data from 3,571 SpA patients and 17,855 matched controls, the prevalence ratio was 1.03 (95% CI 0.85–1.24), indicating no significant difference. This result was consistent across different sexes, age groups, and stricter definitions of COPD, suggesting that SpA patients do not have an increased risk of COPD in the studied region [84].

### 3.5. COPD and Sjögren syndrome

Primary Sjögren’s Syndrome (pSS) is a multifaceted chronic autoimmune disorder marked by dryness of the eyes (keratoconjunctivitis sicca) and mouth (xerostomia), resulting from immune-mediated damage to the lacrimal and salivary glands [85]. In addition, dryness can also impact on other mucosal surfaces, contributing to the clinical presentation known as ‘sicca syndrome’ [86]. When SS manifests in patients already diagnosed with a connective tissue disease, it is referred to as secondary Sjögren’s Syndrome (sSS) [87]. Determining the prevalence and incidence of pSS varies due to differences in diagnostic criteria and study methodologies, hindering the assessment of geographical and temporal patterns [88].

The pathogenesis of pSS involves an intricate interplay between innate and adaptive immunity, as well as salivary gland epithelial cells. The hallmark of pSS is the hyperactivation of B-cells mediated by T-cells [89]. B-cells play diverse roles in the disease pathogenesis, including antigen presentation, autoantibodies production and cytokine secretion [90].

In 30 to 40% of pSS cases, extraglandular sites are affected [91]. Although pulmonary involvement isnot a typically predominant feature of pSS, it can be severe in some instances, ILD representing the most serious complication [92].

In addition to ILD, recent studies (Table 5) have demonstrated an association between COPD and pSS, highlighting the common autoimmune mechanisms involved in both conditions [97]. They are characterized by persistent inflammation in their respective tissues–lungs in COPD and salivary glands in pSS–resulting in tissue damage and clinical symptoms [98,99]. Both conditions involve chronic immune dysregulation with aberrant B-cell activation. In pSS, this leads to the production of anti-Ro and anti-La autoantibodies, while in COPD, emerging evidence suggests that autoantibody formation – potentially driven by neoantigen exposure from oxidative stress or extracellular matrix degradation – may amplify local inflammation and contribute to airway remodeling [100,101].

**Table 5.** Characteristics of selected studies on COPD and Sjögren syndrome.

Reference	Study design	Patients	Outcome/conclusions
Strevens Bolmgren et al. [93] 2017	Cross-sectional study	51 patients with pSS versus 80 controls	- 41% of patients with pSS met the GOLD criteria for COPD. - COPD presence did not significantly influence the respiratory symptoms reported by pSS patients.
Shen et al. [94] 2015	Retrospective study	3.013 females with pSS and 12,052 controls	- Adult women with pSS are at a higher risk of developing COPD compared to those without SS. - The incidence of COPD in the pSS group was 1.4 times higher than in the non-SS group (*p* = 0.007).
Nilsson et al. [95] 2015	Cross-sectional study	51 patients with pSS versus 456 controls	- 41% of pSS patients and 30% of those who have never smoked met the GOLD criteria for COPD.
Mandl et al. [96] 2012	Prospective study	41 female patients with pSS	- At follow-up, 37% of patients with pSS were diagnosed with COPD. - COPD prevalence was higher among pSS patients who had a history of smoking. - These patients showed mainly obstructive pulmonary disease, with some restrictive features.

COPD: Chronic obstructive pulmonary disease; GOLD: Global Initiative for Chronic Obstructive Lung Disease; pSS: Primary Sjögren’s syndrome, SS: Sjögren’s syndrome.

Mandl et al. [96] were the first to investigate the association between COPD and pSS, showing a 37% COPD prevalence in pSS patients over an 11-year observation period. This was also confirmed by Shen et al. [94] who confirmed COPD incidence was 1.4 times higher in pSS over an average follow-up of 7.99 years.

Despite the increased prevalence of COPD in pSS, Bolmgren et al. [83] showed that it does not impact on the severity of symptoms based on St. George’s Respiratory Questionnaire.

However, considering lung disease is linked to reduced survival rates in pSS patients, there is ongoing debate about whether pulmonary function tests (PFTs) should be reserved for pSS patients with respiratory symptoms or conducted routinely in all pSS patients [93].

In the initial study, patients were evaluated through PFTs due to existing respiratory symptoms. However, in a study conducted by Nilsson et al. [95], PFTs were conducted on all patients diagnosed with pSS, regardless of the presence or absence of respiratory symptoms to determine the prevalence of COPD in this population. The authors concluded that COPD is common among patients with pSS, irrespective of smoking status, and accordingly recommended routine PFTs for this patient population. However, current guidelines do not provide explicit recommendations in this regard [102].

### 3.6. COPD and psoriatic arthritis

Psoriasis is an immune-mediated inflammatory skin disease with a complex, multifactorial pathogenesis and it is associated with multiple comorbidities [103]. Its pathophysiology involves interactions between rapidly proliferating keratinocytes and infiltrating, activated immune cells [104]. Psoriatic lesions contain Th1 and Th17 polarized T-cells. Additionally, macrophages, innate immune cells, and endothelial cells infiltrate the psoriatic skin, contributing to the disease’s evolution [105].

Psoriatic arthritis (PsA) is relatively uncommon, affecting approximately 0,1% of the general population [106]. While not as widespread, approximately 30% of psoriasis patients may develop PsA, particularly cases suffering from severe psoriasis or involvement of the nails or scalp [107].

Given the inflammatory nature of psoriasis, PsA, and COPD, and the potential for abnormal immune responses to exacerbate or trigger other inflammatory conditions, the association between them has been studied over time [108]. The idea that a shared cytokine-driven inflammatory process might cause one autoimmune disease to trigger another is slowly gaining recognition [109]. Both psoriasis and COPD share common elements in their pathophysiology, including the involvement of pro-inflammatory cytokines, chemokines, adhesion molecules, and proteases [20,110].

Several studies confirmed that patients with psoriasis are in higher risk of developing COPD [111,112] with an increased predilection for female patients [113].

The pathophysiological connection between the two conditions was also highlighted by their similar responses to the same class of medications. Phosphodiesterases-4 (PDE4) is widely present in cells and plays a key role in the hydrolysis of cyclic adenosine monophosphate, being involved in various inflammatory diseases [114]. So far, four PDE4 inhibitors have been authorized namely roflumilast for COPD and asthma and apremilast for plaque psoriasis and PsA [115]. It is plausible that targeting PDE4 could benefit both patients groups. This approach might facilitate dual screening for these conditions, encourage multidisciplinary involvement in managing PsA, and enable more personalized treatment strategies [116].

### 3.7. COPD and antiphospholipid antibody syndrome

Antiphospholipid syndrome (APS) is a thrombo-inflammatory condition affecting up to a third of SLE cases. While secondary APS is highly prevalent, it may also occur independently [117].

APS is characterized by recurrent venous or arterial thromboses and pregnancy complications in the presence of antiphospholipid antibodies (aPL) represented by lupus anticoagulant, anti-β2-glycoprotein I and/or anti-cardiolipin antibodies [118]. While the APS pathogenesis was initially focused on aPL’s disruption of coagulation and fibrinolysis, it is currently known to involve complex interactions between inflammation and thrombosis [119].

Although venous thrombosis most commonly occurs in the lower limbs and arterial thrombosis in the cerebral arterial circulation, they may be affected by any organ [120]. In rare cases, a severe form of multiorgan thrombosis called catastrophic APS may develop [121].

The range of clinical manifestations linked to aPL positivity is continually expanding, encompassing various lung diseases [122]. Common pulmonary manifestations include thromboembolism and PH, whereas diffuse alveolar hemorrhage, acute respiratory distress syndrome, and pulmonary fibrosis are rare [123].

Although APL can present with pulmonary involvement and can be associated with other autoimmune diseases, there are currently no reported cases in the literature linking APL with COPD. This is likely due to the different pathophysiological mechanisms underlying each condition.

### 3.8. COPD and polymyalgia rheumatica

Polymyalgia rheumatica (PMR) is the most common inflammatory rheumatic condition in the elderly, marked by bilateral shoulder pain and possible neck and hip involvement [124]. PMR shares similar pathophysiological mechanisms with giant cell arteritis, occurring particularly in individuals over 50 years, predominantly females. Diagnosis is primarily clinical, supported by laboratory evidence of acute phase reactions [125]. Researchers have proposed diagnostic criteria, including 2012 European League Against Rheumatism/American College of Rheumatology provisional classification criteria for PMR [126].

Even though there is no specific antibody for PMR, it is now recognized as an autoimmune condition, with research indicating that genetic factors and possibly environmental triggers, particularly viruses might contribute to this autoimmune response [127,128].

The relationship between PMR and lung involvement has not been extensively studied over time, with the limited available data indicating an association between PMR and ILD [129]. Moreover, when the two conditions are present simultaneously, further investigation for a connective tissue disease is recommended [130]. PMR-like symptoms may also occur as a paraneoplastic manifestion in different cancer forms, especially lung cancer [131,132].

Until now, there is no evidence in the literature suggesting any association between PMR and obstructive ventilatory dysfunction, including COPD.

## 4. COPD and inflammatory bowel diseases

Inflammatory bowel diseases (IBDs), Crohn’s disease (CD) and ulcerative colitis (UC), are characterized by chronic inflammation within the gastrointestinal (GI) tract [133]. IBD is regarded as an idiopathic condition mainly affecting patients in Western countries. It also poses significant health-care costs due to the associated high morbidity, hospital admissions and mortality rates [134]. Over 2 million people are suffering from IBD in Europe with a prevalence of over 0.3% in most countries [135].

Both CD and UC have long been associated with inflammation in sites other than the GI tract. Extraintestinal manifestations (EIMs) include the eyes, joint or skin [136]. The European Crohn’s and Colitis Organisation define EIMs as inflammation outside the gut, depending on the extension or translocation of the immune activity from the intestine or as an independent event sharing common factors with IBD [137]. According to research, 21–41% of patients suffering with IBD will develop EIMs throughout their lifetime [138], but rates of up to 47% have been reported [139]. EIMs may occur prior to IBD onset or shortly after.

The digestive tract and respiratory mucosa share common embryological, structural and physiological characteristics, with overlapping immune and environmental factors affecting both systems. The foregut region of the endoderm is the embryological substrate from which both lung and GI tract are derived. The epithelium and submucosal lymphoid tissue are similar, which may explain similar inflammatory reactions [140]. According to Kinose et al. [141] the genetic substrate involving the GI and respiratory tract has emerged from the co-occurrence of IBD and asthma during childhood. Further subsequent studies have described a connection between nucleotide-binding oligomerization domain-containing protein 2 (NOD2) gene polymorphism playing a role in the progression of both COPD and IBD. NOD2 proteins are involved in the recognition of bacterial antigens, a substrate for immune activation [141]. The lung-gut crosstalk signaling is also thought to be mediated by the immunological imbalance between pro- and anti-inflammatory mechanisms. IL-6 and transforming growth factor-beta (TGF-β) activate the T-helper 17 (Th-17) mediated immune response. A subsequent cascade of IL-17A, IL-17F, IL-21, IL-22, IL-26, TNF-α and neutrophil activation promote and sustain the pro-inflammatory state [142,143]. Th-17 and IL-22 responses are also mediated by exposure to segmented filamentous bacteria. Nonetheless, the microbiota seems to be involved in modulation of the lung inflammation, as antibiotic treatment increases the risk of respiratory infections in rendered mice. On the other hand, murine alveolar macrophages without microbiota indicated lower phagocytic activity and bacterial killing [144].

Smoking is the key risk factor in COPD pathogenesis, and it also increases the risk of Crohn’s disease by 3-fold, however, a lower prevalence has been reported in ulcerative colitis [145]. In COPD patients, the intestinal barrier possesses a higher permeability, associated with abnormal lung function. Conversely, smokers that develop COPD transpose pathophysiological changes in the ileum and colon, with an increased risk of IBD [146].

Generally, IBD patients rarely report respiratory symptoms, but up to 90% of them may have abnormal PFTs [147]. Most frequently, there is a decrease in forced expiratory volume in 1 s (FEV1), Tiffeneau index or the transfer coefficient for carbon monoxide. However, in those reporting respiratory symptoms, airways involvement is most seen. The spectrum of the airways’ disease ranges from bronchiectasis (22% of patients), chronic bronchitis (20%), to asthma, bronchitis or COPD [148]. Several authors have proposed that shared mucosal immunity between the lungs and gastrointestinal tract, along with circulating cytokines from gut inflammation, may lead to subclinical lung damage. This damage manifests as altered spirometric values without noticeable respiratory symptoms. Additionally, early airway inflammation may cause spirometric changes detectable well before symptom onset. Studies have also shown high levels of alveolar lymphocytes in bronchoalveolar lavage from asymptomatic CD patients, suggesting alveolitis. Furthermore, many of these patients experience poor nutritional status, which has also been associated with alterations in spirometry values [149]. Nevertheless, we still lack a complete understanding of the mechanisms behind occult pulmonary involvement in IBD, and it remains unclear whether screening for lung involvement in these patients is necessary [150].

Some authors have evaluated the risk of IBD populations to develop obstructive lung disease (OLD), with their results demonstrating that compared to non-IBD individuals, patients with either CD or UC are more likely to develop COPD (Table 6). Jacobsen et al. [151] included 24,238 patients with IBD, out of which 7.9% were identified as having OLD prior to their IBD diagnosis, corroborating the fact that the IBD-population is 60% more likely to associate OLD (adjusted Odds Ratio (OR) 1.60, 95% Confidence Interval (CI): 1.53–1.67). This was further confirmed by Valentin et al. [152] and Pemmasani et al. [153] who identified OLD in IBD cohorts (3% and 8.7%, respectively). Conversely, researchers evaluated the risk of developing IBD in COPD cohorts, with results pointing to higher incidence rates of IBD in COPD compared to non-COPD populations and further increases as the condition progresses. Furthermore, co-existence of IBD in a COPD population, increases the risk of all-cause mortality and risk of death from digestive tract conditions [148,154–156].

**Table 6.** Characteristics of selected studies on COPD and inflammatory bowel disease.

Reference	Study Design	Patients	Risk	Incidence	Comments
Jacobsen et al. [151] 2024	Prospective, case-control study	24.238 patients with IBD matched 1:10 with non-IBD	Adjusted OR: 1.60 for OLD before IBD onset (95% CI: 1.53–1.67). HR: 1.43 for COPD after IBD onset (95% CI: 1.37–1.49).	Adjusted HR: 1.61 for COPD (95% CI: 1.49–1.74)	IBD patients have a 60% higher risk of OLD before IBD onset and a 40% higher risk after IBD diagnosis.
Valentin et al. [152] 2023	Prospective cross-sectional study	325 patients with IBD	N/R	10 patients diagnosed with COPD	Half of the patients reported respiratory symptoms.
Pemmasani et al. [153] 2022	Retrospective observational cohort	87.506 IBD and 87.506 non-IBD controls	CD patients have a 30% higher risk for COPD compared to UC.	8.7% of COPD incidence in IBD cohort	Obstructive respiratory diseases were the most prevalent in the IBD cohort.
Lee et al. [154] 2019	Retrospective, case-control study	1.303.021 COPD and 6,515,105 controls	Adjusted HR: 1.38 (95% CI: 1.25–1.52). Severe COPD: HR 1.70 (95% CI: 1.27–2.21)	COPD incidence: 9.98 vs 7.18 per 100.000 person-years	Higher incidence and risk of IBD in COPD, increasing with COPD severity.
Vutcovici et al. [148] 2016	Retrospective cohort study	273.208 subjects with COPD	HR for all-cause mortality: 1.23 (95% CI 1.09–1.4).	N/R	Increased mortality and risk of death from digestive conditions in COPD patients with IBD.
Brassard et al. [155] 2015	Retrospective cohort study	143.904 subjects with COPD	N/R	CD: 26.2 per 100.000 person-years; Ulcerative colitis: 17 per 100.000 person-years	Higher incidence of CD and UC in COPD and asthma patients.
Ekbom et al. [156] 2008	Population-based cohort study	1.174.557 total individuals; 180,239 COPD patients	UC: HR 1.83 (95% CI 1.61–2.09). CD: HR 2.72 (95% CI 2.33–3.18).	N/R	Increased risk of Crohn’s disease among first-degree relatives of COPD patients.
Ludvigsson et al. [157] 2012	Prospective, case-control study	10.990 patients with celiac disease, 54.129 controls	OR for COPD before celiac disease diagnosis: 1.22 (95% CI: 1.02–1.46)	COPD incidence: 3.5% in celiac disease vs 2.6% in controls	Higher risk of COPD in patients with celiac disease, both before and after diagnosis.

CI: Confidence interval; COPD: Chronic obstructive pulmonary disease; HR: Hazard ratio; IBD: Inflammatory bowel disease; N/R: Not reported; OLD: Obstructive lung disease; OR: Odds ratio; UC: Ulcerative colitis.

### 4.1. COPD and Crohn’s disease

CD is a systemic inflammatory condition involving the gastrointestinal tract, associating EIMs and other autoimmune disorders. It is caused by a series of genetic and environmental factors, including exposure to air pollution and tobacco smoke. Physicians may be probed to screen for respiratory conditions when patients have specific associated symptoms. Valentin et al. [152] identified that out of 325 patients with IBD (more than half with CD), 31.2% had associated dyspnea and 24% were active smokers. The diagnosis of COPD was established in 10 patients after pneumology consultation. In a study conducted by Jacobsen et al. [151] 7369 patients (30.4%) had a diagnosis of CD. The adjusted OR of developing OLD was 1.60 (95% CI 1.53–1.67), with a slightly higher risk of OLD for CD compared to UC. The data was further confirmed by Pemmasani et al. [153] who reported that 8.7% of the 87,506 IBD patients included, were diagnosed with COPD. In contrast, chronic COPD-associated airway inflammation seems to lead to gastrointestinal implications, as this specific population is at a higher risk of IBD development. Moreover, the higher COPD severity, the higher risk of associated IBD, especially CD. This can be partially explained by chronic tissue hypoxia, including the intestinal mucosa, leading to enterocyte damage and integrity loss, a mechanism sustained by pro-inflammatory cytokines further altering the structure of tight junctions [154]. Brassard et al. [155] observed a higher incidence of CD in the 143,904 patients with COPD, counting for 23.1 per 100,000 person-years, compared to 8.8 per 100,000 person-years for UC. Ekbom et al. [156] not only confirmed similar findings, but also supported the hypothesis that first-degree relatives of COPD patients have a higher risk of developing CD (HR 1.25; 95% CI 1.09–1.43).

### 4.2. COPD and ulcerative colitis

UC similarly to CD, is a systemic inflammatory condition, arising in the colonic mucosa, but with a much higher prevalence comparatively. There are several genetic and environmental factors associated with UC, whereas smoking seems to be a protective one. Smokers have been demonstrated to develop a milder form of disease compared to non-smokers with an increase in disease activity in those that quit smoking [158]. Nevertheless, Vutcovici et al. [148] showcased that 697 patients from the COPD cohort (273,208) developed IBD over a mean follow-up time of 4.63 years. Moreover, it showed that UC, compared to CD, increased the risk of mortality in the COPD cohort. A rise in all-cause mortality which was thought to be the consequence of associated digestive mortality [148]. Moreover, patients of older age are more likely to be diagnosed with UC rather than CD [154], with the highest incidence of UC (24.9 cases per 100,000) in the 60–69 age group [155].

### 4.3. COPD and celiac disease

Celiac disease is an autoimmune condition mostly developed in genetically susceptible individuals, primarily affecting the small bowel in the context of gluten ingestion. The main manifestation is malabsorption, although it may present with a wide variety of gastrointestinal and extraintestinal signs and symptoms [159]. There are several genetic and environmental factors that may lead to an increased COPD incidence among celiac disease. Ludvigsson et al. [157] revealed an increased COPD risk in patients suffering from celiac disease, with 3.5% of them developing COPD (HR 1.17, 95% CI: 1.00–1.38), corresponding to an excess risk of 79/100,000 person-years. Finally, male sex has been associated with a higher risk of celiac disease, although there might be an association with high smoking prevalence. Younger age (40–59 years) seems to be a risk factor, as well, supporting a possible genetic susceptibility [159].

## 5. COPD and nervous system diseases

### 5.1. COPD and multiple sclerosis

Multiple sclerosis (MS) is an autoimmune disorder targeting the central nervous system (CNS), characterized by recurrent episodes of acute neurological dysfunction or a gradual neurological decline. This condition typically manifests with physical impairments in motor skills, sensory perception, balance, or vision [160]. In MS, the body’s immune system erroneously targets the myelin sheath leading to inflammation and subsequent damage. This damage disrupts the normal transmission of electrical impulses along the nerves, resulting in a broad spectrum of neurological symptoms [161]. Owing to the formation of scar tissue (sclerosis) in multiple CNS regions. The immune system’s assault on myelin and oligodendrocytes, subsequently results in demyelination, resulting in the formation of lesions or plaques. Over time, repeated episodes of inflammation and repair can also cause irreversible loss of nerve fibers, namely, axonal damage. The precise cause of MS remains unclear, though it is thought to arise from a combination of genetic predisposition and environmental influences. Research suggests that viral infections, low vitamin D levels, and smoking might also contribute to the risk of developing MS (Table 7) [165,166].

**Table 7.** Characteristics of selected studies on COPD and multiple sclerosis.

Reference	Study design	Patients	Outcome/conclusions
Ashtari et al. [162] 2018	Cross-sectional study	1170 patients	- Air pollution increases the incidence and severity of MS and COPD. - Oxidative stress and vitamin D deficiency are the principal mechanisms.
Marrie et al. [163] 2016	Population-based cohort study	44.452 patients with MS and 220,849 matched controls	- Over 10% of MS patients are affected by CLD, including COPD.
Kang et al. [164] 2010	Population-based controlled study	898 patients with MS	- Patients with MS face a higher risk of multiple medical comorbidities compared to a matched control group.

CLD: Chronic lung disease; COPD: Chronic obstructive pulmonary disease; MS: Multiple sclerosis.

Ghoshouni et al. [167] identified a significant association between MS and COPD. While limited research has been conducted on this relationship, prior studies have presented some evidence to elucidate it.

Burke et al. [168] demonstrated that approximately 70% of COPD patients exhibit some degree of systemic inflammation, as indicated by elevated levels of C reactive protein (CRP), IL-6, IL-8, fibrinogen, TNF-α, and leukocytes. However, only 16% of these patients experience persistent inflammation [169]. Individuals with persistent inflammation, even with similar levels of lung function impairment, experience markedly higher rates of mortality and exacerbations [170]. Moreover, this ongoing systemic inflammatory profile has been linked to cardiovascular comorbidities and mortality, regardless of the presence of COPD [171], while elevated levels of TNF-α have been associated with cachexia and sarcopenia [172].

Oxidative stress plays a role in various pathological processes, such as NF-κB and phosphoinositide 3-kinase pathways in COPD, which have, nowadays, become potential targets for therapeutic interventions. Additionally, as indicated by elevated levels of serum 8-hydroxy-2′-deoxyguanosine, oxidative stress is probably a factor contributing to multimorbidity in COPD [168].

In immunological research, it has been indicated that COPD and MS share common cytokines and inflammatory pathways [173]. Specifically, both diseases exhibit elevated levels of TNF-α, IL-6, and IL-1β, critical in driving the inflammatory processes characteristic of these conditions [174]. In parallel, both MS and COPD involve dysregulated T cell responses, particularly Th17 polarization and autoreactive CD4+ T cells, which may contribute to sustained autoimmunity and target-organ injury [175,176]. Moreover, pathways involving NF-κB and Janus kinase/signal transducer and activator of transcription (JAK/STAT) are implicated in the pathogenesis of both COPD and MS, further contributing to sustained inflammation and tissue damage [177]. This immunological overlap suggests that therapeutic strategies targeting these shared pathways may benefit patients suffering from both diseases, offering new avenues for intervention and management [178].

### 5.2. COPD and Guillain-Barré syndrome

Guillain-Barré Syndrome (GBS) is an acute, rapidly progressing autoimmune disorder affecting the peripheral nervous system. GBS typically occurs following a bacterial or viral infection, with the immune system mistakenly attacking the myelin sheath of peripheral nerves. This demyelination may cause symptoms such as sudden muscle weakness, areflexia, and, in severe cases, paralysis. GBS may lead to progressive weakness of the respiratory muscles, impacting both the inspiratory and expiratory muscles [179].

The typical signs of respiratory failure appear later in the disease progression, with early symptoms being tachypnea, tachycardia, flaccid dysarthria. As the condition worsens, severe diaphragmatic weakness is indicated by the use of orthopnea, paradoxical breathing and accessory respiratory muscles [180]. In approximately 20–30% of patients with GBS, the condition progresses to respiratory failure and autonomical dysfunction, rendering the patients’ admission in neurocritical care absolutely necessary and leading to high rates of mortality [181].

The progression of GBS is often rapid, with symptoms developing from days to weeks, whilst it may become life-threatening if the respiratory muscles are affected. Rehabilitation can vary, with some patients recovering completely and others experiencing long-term effects [182].

In COPD, chronic respiratory infections contribute to the persistent inflammation and immune system activation, marked by increased levels of IL-6 and TNF-α [183]. Similarly, GBS is often preceded by respiratory or gastrointestinal infections, believed to trigger an aberrant immune response leading to an autoimmune attack on peripheral nerves [184].

The shared immunological mechanisms between COPD and GBS include the involvement of molecular mimicry, where microbial antigens resemble host tissue antigens, leading to cross-reactivity and autoimmunity [185]. Additionally, both conditions exhibit increased levels of specific antibodies and immune complexes contributing to tissue damage and disease progression (Table 8) [9].

**Table 8.** Characteristics of selected studies on COPD and GBS.

Reference	Study design	Patients	Outcome/conclusions
Davidson et al. [186] 2021	Cross-sectional study	714 individuals	- Both GBS and COPD result in reduced mobility, increased pain, and psychological distress. - The combined effects of these conditions negatively impact on patients’ overall health and quality of life.
Khanna et al. [180] 2017	Prospective, cross-sectional study	28 patients	- Subacute GBS patients present with a high prevalence of restrictive pulmonary dysfunction associated with reduced chest expansion. - Similar to COPD, both conditions show significant pulmonary impairment.
Aggarwal et al. [187] 2003	Retrospective study	11 patients	- GBS primarily affects the peripheral nervous system, but complications like ventilator-associated pneumonia and systemic infections, common in COPD, can worsen the condition. - The study highlights that pulmonary dysfunction in GBS is frequently seen even in the subacute phase.
Mozhdehipanah et al. [188] 2021	Case series	55-year-old woman with COPD	- The connection between COPD and GBS lies in the compounded health risks and severe complications when both conditions coexist. - This overlap requires vigilant monitoring and integrated care to manage the increased risks effectively.
García-Manzanedo et al. [189] 2020	Case report	77-year-old male with COPD	- Severe neurological manifestations of COVID-19 in patients with preexisting conditions like COPD reduce the focus on comprehensive neurological assessments. - This situation may suggest a potential link between COVID-19 and the onset of GBS.

COPD: Chronic obstructive pulmonary disease; COVID-19: Coronavirus disease 2019; GBS: Guillain–Barré syndrome.

## 6. COPD and pernicious anemia

Pernicious anemia (PA), also referred to as Biermer’s disease, is an autoimmune condition that impairs the body’s ability to absorb vitamin B12, a crucial nutrient for red blood cell production and nervous system function [190]. The immune system either attacks directly the stomach’s parietal cells or the production of intrinsic factor, a glycoprotein necessary for vitamin B12 absorption [191]. This process leads to a deficiency in vitamin B12, resulting in bone marrow disorders, with the production of macrocytic, dysfunctional red blood cells and various neurological defects. Common symptoms include fatigue, pallor, shortness of breath, tingling and paresthesia [192]. If left untreated, PA may lead to serious complications, including subacute combined degeneration of the spinal cord, optic nerve neuropathy, sensorimotor polyneuropathy and defective cognition [191].

In the context of chronic inflammation, both COPD and PA involve persistent inflammatory processes [193]. Despite both conditions sharing systemic presentations, chronic inflammation in COPD is localized in the respiratory tract, while in PA, it is primarily gastrointestinal. Research now suggests (Table 9) that the chronic inflammatory milieu in COPD could potentially exacerbate the autoimmune processes in PA and vice versa, significantly worsening patient prognosis [197].

**Table 9.** Characteristics of selected studies on COPD and pernicious anemia.

Reference	Study design	Patients	Outcome/conclusions
Lee et al. [194] 2019	Cohort follow-up study	265,459 healthy participants	- COPD patients were not significantly associated with anemia in chronic diseases.
Kovchun et al. [195] 2020	Observational study	202 patients	- PA involves vitamin B_12_ deficiency, leading to anemia and elevated mean corpuscular volume. - Anemia in COPD involves inflammation-related mechanisms.
Takahashi et al. [192] 2016	Observational study	628 patients	- PA is characterized by macrocytosis. - Macrocytic anemia may include hypoxemia associated with COPD.
Tsantes et al. [196] 2004	Observational study	32 COPD diagnosed patients	- Macrocytosis in COPD is linked to acute erythropoietic stress from frequent exacerbations. - PA may result from this stress response.

COPD: Chronic obstructive pulmonary disease; PA: Pernicious anemia.

Autoimmunity plays a significant role in both diseases. The dysregulated immune response in COPD may contribute to the breakdown of immune tolerance observed in PA, while the systemic inflammation and immune activation in pernicious anemia could potentially influence the immune landscape in COPD [194].

Moreover, the pro-inflammatory cytokines elevated in COPD, such as TNF-α and IL-6, are also believed to have an involvement in PA [198]. These cytokines promote chronic inflammation and tissular damage, perpetuating the pathogenic processes in both conditions.

## 7. COPD and type 1 diabetes mellitus

Type 1 diabetes mellitus (T1DM) is a chronic autoimmune condition, characterized by the destruction of the insulin-producing pancreatic beta cells, tasked with insulin production [199]. In the absence of insulin, hyperglycemia occurs, leading to polyuria, polydipsia, unintended weight loss, and fatigue [200]. Long-term complications of uncontrolled T1DM may encompass target organ damage, namely cardiovascular, renal, ocular and cerebral. Therapeutic management of T1DM involves lifelong insulin therapy with regular glycemic monitoring, and a balanced diet [201].

The interplay between COPD and T1DM (Table 10) involves complex metabolic and immunological interactions [207]. COPD is characterized by chronic inflammation associated with elevated levels of CRP, TNF-α, and IL-6. These inflammatory markers not only contribute to the progression of COPD but may also induce systemic complications, interfering with insulin signaling and glucose metabolism [208]. Conversely, the autoimmune nature of T1DM, which involves the immune-mediated destruction of pancreatic beta cells, may further exacerbate systemic inflammation, potentially worsening the inflammatory milieu in COPD. Shared molecular mechanisms may further strengthen this link. In COPD, oxidative stress and chronic lung inflammation lead to the formation of modified self-antigens, such as carbonylated proteins and elastin fragments, which can trigger autoreactive immune responses involving Th1 and Th17 cells. These mechanisms resemble those driving pancreatic beta-cell destruction in T1DM, where IL-1β, IL-6, and TNF-α play central roles [209–211]. Both diseases activate key inflammatory pathways such as NF-κB and NLRP3 inflammasome signaling, contributing to sustained immune dysregulation [212]. Moreover, alpha-1 antitrypsin (AAT) deficiency – associated with COPD – has been implicated in modulating immune tolerance, and exogenous AAT administration has shown protective effects in animal models of T1DM, suggesting a possible immunological bridge between the two conditions [213,214].

**Table 10.** Characteristics of selected studies on COPD and type 1 diabetes.

Reference	Study design	Patients	Outcome/conclusions
Crisafulli et al. [202] 2014	Cohort-based study	125 patients admitted for an acute COPD exacerbation	- T1DM is a key predictor of early readmission in COPD patients.
Ehrlich et al. [203] 2010	Retrospective cohort study	77,637 patients with diabetes	- T1DM patients have a significantly increased risk of developing COPD. - Higher glycated hemoglobin levels further increase this risk.
O’Byrne et al. [204] 2012	Pooled analysis	8259 patients with COPD at risk of onset of new diabetes mellitus	- Inhaled corticosteroids in asthma or COPD patients do not significantly increase the risk of new-onset diabetes mellitus. - No significant increase in hyperglycemia compared to placebo or non-inhaled corticosteroid treatments.
Caughey et al. [205] 2013	Retrospective study	18,226 subjects with diabetes of which 5.9% had COPD	- Patients with both diabetes and COPD have an increased risk of hospitalization for diabetes-related complications. - Increased risk occurs only with high doses of corticosteroids.
Ho et al. [206] 2017	Observational study	2,015 individuals	- Impaired lung function in diabetes mellitus patients may result from direct exposure to hyperglycemia. - Diabetes mellitus exacerbates mortality and survival outcomes in COPD patients. - Pre-existing diabetes mellitus increases mortality risk in COPD patients.

COPD: Chronic obstructive pulmonary disease; T1DM: Type 1 diabetes mellitus.

This bidirectional relation suggests that for effective management, a comprehensive approach that addresses both metabolic and immunological factors is required [215]. Comprehending these interactions is essential for creating targeted therapeutic approaches that can alleviate the negative impacts of chronic inflammation on glycemic control and vice versa, ultimately improving the quality of life for patients affected by both conditions [206].

## 8. COPD and thyroid dysfunction

Thyroid dysfunctions are frequently observed in patients with COPD, with hypothyroidism being the most prevalent. The diagnosis of thyroid disease in this group of patients can be challenging due to symptom overlap with COPD, including dyspnea, fatigue, weight loss, and decreased appetite [216]. Several mechanisms have been proposed to elucidate the relationship between hypothyroidism and COPD. Inflammatory processes inherent to COPD are believed to stimulate the production of cytokines such as interleukin-1, interleukin-6, and tumor necrosis factor-alpha. These cytokines are known to inhibit the synthesis and secretion of thyroid-stimulating hormone (TSH) and impair the peripheral conversion of thyroxine (T4) to triiodothyronine (T3) [217]. Additionally, smoking promotes respiratory inflammation and reduces the bioavailability of thyroid hormones [218]. Dimopoulou et al. [219] demonstrated that hypoxemic patients with a forced expiratory volume in one second (FEV1) of less than 50% exhibit diminished T4 to T3 conversion. Furthermore, hypothyroidism is associated with decreased muscular strength, which may exacerbate COPD severity [218]. In addition, COPD may share autoimmune features with thyroid dysfunction. Circulating anti-thyroid antibodies such as anti-TPO and anti-thyroglobulin have been identified in COPD patients, even in the absence of clinical thyroid disease, suggesting a possible immunological link between the lungs and thyroid gland. Autoimmune thyroiditis and COPD may therefore coexist as part of a broader dysregulation of immune tolerance [7].

Although elevated TSH levels are correlated with increased rates of COPD exacerbations, hypothyroidism does not appear to significantly impact the quality of life in COPD patients [217]. Hyperthyroidism may induce hyperventilation by amplifying sensitivity to hypoxic and hypercapnic stimuli. Additionally, increased catabolic activity in hyperthyroidism often results in significant muscular weakness [218]. Furthermore, an autoimmune mechanism has also been suggested. In a study conducted by Fatih et al. [220], hyperthyroid patients demonstrated elevated CRP levels, which were negatively correlated with TSH levels. More importantly, raising free T3 has been associated with worse COPD outcomes and is now considered a prognostic marker [218]. All in all, both hypothyroidism and hyperthyroidism have a significant association with COPD, affecting respiratory function and exacerbating COPD severity (Table 11). Addressing these thyroid abnormalities may improve clinical outcomes, however, further research is required to elucidate the underlying pathophysiological mechanisms and optimize treatment strategies.

**Table 11.** Characteristics of selected studies on COPD and thyroid dysfunctions.

Reference	Study Design	Patients	Outcomes/conclusions
El-Yazed et al. [221] 2013	Comparative cross-sectional study	50 COPD patients and 50 healthy smokers	- Free T3 levels elevated in COPD patients. - Free T3 increased with disease severity. - Negative correlation with PaO_2_, SO_2_, and pulmonary function tests.
Singh et al. [222] 2016	Prospective cohort study	201 COPD patients	- 64.6% of COPD patients had thyroid disorders. - 59.2% hypothyroidism, 5.4% hyperthyroidism. - No statistically significant association (*p* = 0.213).
Dimopoulou et al. [219] 2001	Cross-sectional study	26 patients with mild-to-moderate COPD, 20 patients with severe COPD	- T3/T4 ratio positively correlated with PaO_2_ in severe COPD. - No significant correlation between thyroid hormones and other variables.
Gumus et al. [223] 2021	Cross-sectional study	309 COPD patients	- 22% of COPD patients had thyroid disorders (2% with hypothyroidism, 15% with euthyroidism, 5% with hyperthyroidism).
Huang et al. [224] 2021	Cross-sectional study	97 patients with moderate-to-severe COPD and 37 patients with very severe COPD	- Hospitalization duration negatively correlated with TSH levels (*p* = 0.003). - 26.87% had thyroid dysfunctions.
Sebasan et al. [225] 2021	Cross-sectional study	36 patients with moderate COPD, 14 patients with severe COPD	- 47.2% of moderate COPD patients had hypothyroidism. - 71.4% of severe COPD patients had hypothyroidism.
Terzano et al. [226] 2013	Cross-sectional study	189 COPD patients	- 33% had hypothyroidism. - 22% had subclinical hypothyroidism. - 48% had hyperthyroidism. - Hypothyroid patients had lower pO_2_ and MEP values. - TSH levels negatively correlated with PaO_2_ and MEP.
Ulasli et al. [227] 2013	Prospective cohort study	44 COPD patients with hypothyroidism, 44 COPD patients with normal thyroid function, and 40 healthy controls	- Strong positive correlation between TSH levels and exacerbation frequency (*p* < 0.0001).

COPD: Chronic obstructive pulmonary disease; MEP: Maximal expiratory pressure; PaO_2_: Partial pressure of oxygen; SO_2_: Oxygen saturation; TSH: Thyroid stimulating hormone.

The table below provides a concise summary of the previously presented findings, illustrating the potential mechanisms underlying the association between systemic autoimmune diseases and COPD (Table 12).

**Table 12.** Mechanistic pathways potentially linking systemic autoimmune diseases to COPD.

Autoimmune Disease	Potential Mechanisms Linking to COPD
Rheumatoid Arthritis	- Increased risk of COPD in seropositive RA due to smoking-induced inflammation and production of ACPA [46]. - Dysbiosis leading to IL-17 production, triggering pro-inflammatory cytokines (IL-1β, IL-6, and IL-23). [50,51]
Systemic Lupus Erythematosus	- Production of autoantibodies, particularly ANA, which may increase with COPD progression, potentially predisposing to SLE [72] - Smoking is a common risk factor exacerbating both conditions [68]. - Lung involvement in SLE can manifest as pleuritis, pulmonary hypertension, and infections that complicate COPD [65,66].
Systemic Sclerosis	- Chronic inflammation and fibrosis in scleroderma may exacerbate airway obstruction seen in COPD [76]. - Smoking and environmental factors can worsen the lung condition in both diseases [77]. - Increased mortality risk in patients with both scleroderma and COPD, particularly in the context of pulmonary hypertension [79].
Axial Spondyloarthritis	- Shared risk factors, especially smoking, contributing to lung injury [82]. - Chronic systemic inflammation leading to lung damage [82].
Sjögren Syndrome	- Shared B-cell hyperactivation and production of autoantibodies affecting lung tissues [89,100,101].
Psoriatic Arthritis	- Inflammatory cytokine pathways common to both conditions. [20,110]. - Shared genetic and environmental factors contributing to both diseases. [111]. - Abnormal immune responses exacerbating lung inflammation [109].
Antiphospholipid Antibody Syndrome	No evidence suggesting a potential interaction.
Polymyalgia Rheumatica	No evidence suggesting a potential interaction.
Crohn’s Disease and Ulcerative Colitis	- Altered gut-lung axis with immune activation causing lung inflammation [141]. - Chronic tissue hypoxia from COPD leading to intestinal mucosal damage and increased IBD risk [146]. - Smoking is a risk factor for both conditions.
Celiac Disease	- Genetic susceptibility leads to increased COPD risk among patients with celiac disease [157]. - Smoking is a common risk factor for both diseases [179].
Multiple Sclerosis	- Both conditions exhibit elevated levels of pro-inflammatory cytokines (TNF-α, IL-6, IL-1β), contributing to chronic inflammation and tissue damage [185]- COPD-related oxidative stress (elevated serum 8-hydroxy-2′-deoxyguanosine) may exacerbate MS pathology, leading to increased inflammation [185].- NF-κB and JAK/STAT pathways are implicated in the pathogenesis of both diseases, contributing to inflammation and demyelination in MS, and lung damage in COPD. [193]
Guillain-Barré Syndrome	- Respiratory infections in COPD may trigger an autoimmune response through molecular mimicry, similar to GBS. [185]- Chronic respiratory infections can activate the immune system, leading to increased cytokines (TNF-α, IL-6), involved in both GBS and COPD. [183]- Both COPD and GBS show elevated immune complexes and antibodies that contribute to autoimmunity and tissue damage. [183,184]
Pernicious Anemia	- Both conditions are characterized by chronic inflammation [193].- Pro-inflammatory cytokines (TNF-α, IL-6) elevated in COPD may promote inflammation in PA [198].
Type 1 Diabetes Mellitus	- Chronic inflammation in COPD (CRP, TNF-α, IL-6) can disrupt insulin signaling and glucose metabolism [208]. - The autoimmune destruction of pancreatic beta cells in T1DM may worsen systemic inflammation in COPD [215].
Thyroid conditions	- Inflammation in COPD (IL-1, IL-6, TNF-α) impairs thyroid hormone regulation [217]. - Smoking in COPD reduces thyroid hormone bioavailability [218]. - Hypothyroidism decreases muscular strength, worsening COPD severity [218]. - Hyperthyroidism induces hyperventilation, exacerbating COPD symptoms [218,220]. - Hypercatabolic activity in hyperthyroidism weakens respiratory muscles, worsening COPD [218].

ACPA: Anti-citrullinated protein antibody, ANA: Anti-nuclear antibody, COPD: Chronic obstructive pulmonary disease, CRP: C-reactive protein, GBS: Guillain-Barre syndrome, IL-1β: Interleukin-1 beta, IL-17: Interleukin-17, IL-6: Interleukin-6, IL-23: Interleukin-23, JAK/STAT: Janus kinase/signal transducer and activator of transcription, MS: Multiple sclerosis, NF-κB: Nuclear factor kappa-light-chain-enhancer of activated B-cells, SLE: Systemic lupus erythematosus, TNF-α: Tumor necrosis factor-alpha.

## 9. Conclusions

COPD is a multifactorial disease, primarily driven by airflow limitation and lung tissue destruction, which has long been associated with smoking. However, emerging evidence suggests a significant role for systemic inflammation and autoimmune processes in its pathogenesis. This review highlights how mechanisms such as oxidative stress, protein carbonylation, citrullination, and immune activation link COPD to autoimmune conditions such as rheumatoid arthritis, inflammatory bowel diseases, neurological disorders, pernicious anemia, type 1 diabetes mellitus and thyroid dysfunctions. These shared pathways, including the formation of neoantigens and the triggering of both innate and adaptive immune responses, contribute to chronic inflammation, lung tissue destruction, and the progressive decline in lung function typical of COPD.

Recent findings underscore the pivotal role of key cytokines and chemokines – such as IL-17, TNF-α, IL-6, BAFF, and CXCL13 – as well as specific autoantibodies (e.g. ACPA, ANA) that not only drive local inflammation but may also perpetuate systemic autoimmunity in susceptible individuals. The bidirectional relationships described here, alongside shared risk factors such as smoking and microbiome dysbiosis, emphasize that COPD should be considered within the broader context of immune-mediated diseases. Importantly, this intersection raises relevant clinical questions. As monoclonal antibody therapies targeting cytokines or B-cell pathways (e.g. anti-TNF agents, anti-IL-17, anti-BAFF therapies) have transformed the management of autoimmune conditions, future studies should assess whether these biologics might benefit subgroups of COPD patients with strong autoimmune phenotypes. While some therapeutic overlaps – such as PDE4 inhibitors in both COPD and psoriasis – may already exist, the role of targeted immunomodulation in COPD remains underexplored. Going forward, more robust translational and clinical research is needed to elucidate these shared mechanisms, refine phenotyping of patients with overlapping pulmonary and systemic autoimmune features, and evaluate the safety and efficacy of immunotherapies that could potentially modify disease course. Additionally, greater attention to the role of autoantibodies as diagnostic, prognostic, or therapeutic biomarkers may open new avenues for personalized approaches in COPD care.

In conclusion, recognizing COPD as part of the continuum of immune-mediated diseases not only reshapes our understanding of its pathogenesis but also holds promise for future integrated strategies in prevention, diagnosis, and treatment.

## Data Availability

Data sharing is not applicable to this article as no data were created or analysed in this review article.

## References

[1] Boers E, Barrett M, Su JG, et al. Global burden of chronic obstructive pulmonary disease through 2050. JAMA Netw Open. 2023;6(12):e2346598. doi:10.1001/jamanetworkopen.2023.46598. 38060225 PMC10704283

[2] Shaykhiev R, Ronald CG. Innate immunity and chronic obstructive pulmonary disease: a mini-review. Gerontology. 2013;59(6):481–489. doi:10.1159/000354173. 24008598 PMC3833667

[3] Ni L, Dong C. Roles of myeloid and lymphoid cells in the pathogenesis of chronic obstructive pulmonary disease. Front Immunol. 2018;9:1431. doi:10.3389/fimmu.2018.01431. 29977245 PMC6021485

[4] Corlateanu A, Plahotniuc A, Corlateanu O, et al. Multidimensional indices in the assessment of chronic obstructive pulmonary disease. Respir Med. 2021;185:106519. doi:10.1016/j.rmed.2021.106519. 34175803

[5] Kotlyarov S. The role of smoking in the mechanisms of development of chronic obstructive pulmonary disease and atherosclerosis. Int J Mol Sci. 2023;24(10):8725. doi:10.3390/ijms24108725. 37240069 PMC10217854

[6] van Eeden SF, Hogg JC. Immune-modulation in chronic obstructive pulmonary disease: current concepts and future strategies. Respiration. 2020;99(7):550–565. doi:10.1159/000502261. 31480060

[7] Packard TA, Li QZ, Cosgrove GP, et al. COPD is associated with production of autoantibodies to a broad spectrum of self-antigens, correlative with disease phenotype. Immunol Res. 2013;55(1–3):48–57. doi:10.1007/s12026-012-8347-x. 22941590 PMC3919062

[8] Błach J, Siedliński M, Sydor W. Immunology in COPD and the use of combustible cigarettes and heated tobacco products. Eur J Med Res. 2023;28(1):397. doi:10.1186/s40001-023-01374-2. 37794516 PMC10548761

[9] Dong LL, Liu ZY, Chen K-J, et al. The persistent inflammation in COPD: Is autoimmunity the core mechanism? Eur Respir Rev. 2024;33(171):230137. doi:10.1183/16000617.0137-2023. 38537947 PMC10966473

[10] Zhou J-S, Li Z-Y, Xu X-C, et al. Cigarette smoke-initiated autoimmunity facilitates sensitisation to elastin-induced COPD-like pathologies in mice. Eur Respir J. 2020;56(3):2000404. doi:10.1183/13993003.00404-2020. 32366484

[11] Nagaraja V, Mira-Avendano I, Diaz-Arumir A, et al. Interstitial lung disease in autoimmune diseases. Revista Colombiana De Reumatología. 2024;31:S139–S153. doi:10.1016/j.rcreu.2023.12.004. PMC11376317 39238598

[12] Atienza-Mateo B, Remuzgo-Martínez S, Mora Cuesta VM, et al. The spectrum of interstitial lung disease associated with autoimmune diseases: data of a 3.6-year prospective study from a referral center of interstitial lung disease and lung transplantation. J Clin Med. 2020;9(6):1606. doi:10.3390/jcm9061606. 32466389 PMC7356573

[13] Seys LJM, Verhamme FM, Schinwald A, et al. Role of B cell–activating factor in chronic obstructive pulmonary disease. Am J Respir Crit Care Med. 2015;192(6):706–718. doi:10.1164/rccm.201501-0103OC. 26266827

[14] Chen J, Wang T, Li X, et al. DNA of neutrophil extracellular traps promote NF-κB-dependent autoimmunity via cGAS/TLR9 in chronic obstructive pulmonary disease. Signal Transduct Target Ther. 2024;9(1):163. doi:10.1038/s41392-024-01881-6. 38880789 PMC11180664

[15] Ueno M, Maeno T, Nishimura S, et al. Alendronate inhalation ameliorates elastase-induced pulmonary emphysema in mice by induction of apoptosis of alveolar macrophages. Nat Commun. 2015;6(1):6332. doi:10.1038/ncomms7332. 25757189

[16] Kirkham PA, Caramori G, Casolari P, et al. Oxidative stress–induced antibodies to carbonyl-modified protein correlate with severity of chronic obstructive pulmonary disease. Am J Respir Crit Care Med. 2011;184(7):796–802. doi:10.1164/rccm.201010-1605OC. 21965015 PMC3398415

[17] Zinellu E, Zinellu A, Giuseppe A, et al. Circulating biomarkers of oxidative stress in chronic obstructive pulmonary disease: a systematic review. Respir Res. 2016;17(1):150. doi:10.1186/s12931-016-0471-z. 27842552 PMC5109807

[18] Lugli EB, Correia RESM, Fischer R, et al. Expression of citrulline and homocitrulline residues in the lungs of non-smokers and smokers: implications for autoimmunity in rheumatoid arthritis. Arthritis Res Ther. 2015;17(1):9. doi:10.1186/s13075-015-0520-x. 25600626 PMC4349479

[19] Kotlyarov S. Analysis of differentially expressed genes and signaling pathways involved in atherosclerosis and chronic obstructive pulmonary disease. Biomol Concepts. 2022;13(1):34–54. 21no doi:10.1515/bmc-2022-0001. 35189051

[20] Chung KF, Adcock IM. Multifaceted mechanisms in COPD: inflammation, immunity, and tissue repair and destruction. Eur Respir J. 2008;31(6):1334–1356. doi:10.1183/09031936.00018908. 18515558

[21] de Bont CM, Boelens WC, Pruijn GJM. NETosis, complement, and coagulation: a triangular relationship. Cellul Molecu Immunol. 2019;16(1):19–27. doi:10.1038/s41423-018-0024-0. PMC6318284 29572545

[22] Shen Y, Chen L, Chen J, et al. Mitochondrial damage-associated molecular patterns in chronic obstructive pulmonary disease: pathogenetic mechanism and therapeutic target. J Transl Int Med. 2023;11(4):330–340. doi:10.2478/jtim-2022-0019. 38130648 PMC10732348

[23] Kang N, Liu X, Haneef K, et al. Old and new damage-associated molecular patterns (DAMPs) in autoimmune diseases. Rheumatol Autoimmun. 2022;2(4):185–197. doi:10.1002/rai2.12046.

[24] Naessens T, Morias Y, Hamrud E, et al. Human lung conventional dendritic cells orchestrate lymphoid neogenesis during chronic obstructive pulmonary disease. Am J Respir Crit Care Med. 2020;202(4):535–548. doi:10.1164/rccm.201906-1123OC. 32255375 PMC7616955

[25] Freeman CM, Curtis JL. Lung dendritic cells: shaping immune responses throughout COPD progression. Am J Respir Cell Mol Biol. 2016;56(2):152–159. doi:10.1165/rcmb.2016-0272TR. PMC6222925 27767327

[26] Lee DSW, Rojas OL, Gommerman JL. B Cell depletion therapies in autoimmune disease: advances and mechanistic insights. Nat Rev Drug Discov. 2021;20(3):179–199. doi:10.1038/s41573-020-00092-2. 33324003 PMC7737718

[27] Hardavella G, Tzortzaki EG, Siozopoulou V, et al. Lymphangiogenesis in COPD: another link in the pathogenesis of the disease. Respir Med. 2012;106(5):687–693. doi:10.1016/j.rmed.2011.11.011. 22154125

[28] Bracke KR, D’hulst AI, Maes T, et al. Cigarette smoke-induced pulmonary inflammation and emphysema are attenuated in CCR6-deficient mice. J Immunol. 2006;177(7):4350–4359. doi:10.4049/jimmunol.177.7.4350. 16982869

[29] Lee S-H, Goswami S, Grudo A, et al. Antielastin autoimmunity in tobacco smoking–induced emphysema. Nat Med. 2007;13(5):567–569. doi:10.1038/nm1583. 17450149

[30] van der Strate BWA, Postma DS, Brandsma C-A, et al. Cigarette smoke–induced emphysema. Am J Respir Crit Care Med. 2006;173(7):751–758. doi:10.1164/rccm.200504-594OC. 16399994

[31] Alvarado A. Autoimmunity in chronic obstructive pulmonary disease: un update. Clin Res Trial. 2018;4(3):222. doi:10.15761/CRT.1000222.

[32] Qi Y, Yan Y, Tang D, et al. Inflammatory and immune mechanisms in COPD: current status and therapeutic prospects. J Inflamm Res. 2024;17:6603–6618. doi:10.2147/JIR.S478568. 39318994 PMC11421452

[33] Olloquequi J, Montes JF, Prats A, et al. Significant increase of CD57^+^cells in pulmonary lymphoid follicles of COPD patients. Eur Respir J. 2011;37(2):289–298. doi:10.1183/09031936.00201509. 20525712

[34] Yamada H, Kaibara N, Okano S, et al. Interleukin-15 selectively expands CD57 + CD28 − CD4+ T cells, which are increased in active rheumatoid arthritis. Clin Immunol. 2007;124(3):328–335. doi:10.1016/j.clim.2007.06.001. 17644042

[35] Witkop EM, Diggins K, Wiedeman A, et al. Interconnected lineage trajectories link conventional and natural killer (NK)-like exhausted CD8+ T cells beneficial in type 1 diabetes. Commun Biol. 2024;7(1):773. doi:10.1038/s42003-024-06456-3. 38937521 PMC11211332

[36] Jiang J, Yang M, Zhu H, et al. CD4^+^CD57^+^ senescent T cells as promoters of systemic lupus erythematosus pathogenesis and the therapeutic potential of senolytic BCL-2 inhibitor. Eur J Immunol. 2024;54(7):e2350603. doi:10.1002/eji.202350603. 38752316

[37] Paldino G, Tedeschi V, Proganò V, et al. An immunosenescent CD8+ T cell subset in patients with axial Spondyloarthritis and Psoriatic Arthritis links spontaneous motility to telomere shortening and dysfunction. Arthrit Rheumatol. 2025;77(7):854–866. doi:10.1002/art.43109. PMC12209751 39835465

[38] Moutsopoulos HM. Autoimmune rheumatic diseases: One or many diseases? J Transl Autoimmun. 2021;4:100129. doi:10.1016/j.jtauto.2021.100129. 35005593 PMC8716565

[39] Ma Y, Tong H, Zhang X, et al. Chronic obstructive pulmonary disease in rheumatoid arthritis: a systematic review and meta-analysis. Respir Res. 2019;20(1):144. doi:10.1186/s12931-019-1123-x. 31288799 PMC6617695

[40] Yao Y, Zhou J, Diao X, et al. Association between tumor necrosis factor-α and chronic obstructive pulmonary disease: a systematic review and meta-analysis. Ther Adv Respir Dis. 2019;13:1753466619866096. doi:10.1177/1753466619866096. 31390957 PMC6688146

[41] Aslani MR, Amani M, Moghadas F, et al. Adipolin and IL-6 serum levels in chronic obstructive pulmonary disease. Adv Respir Med. 2022;90(5):391–398. doi:10.3390/arm90050049. 36136851 PMC9717330

[42] P, Wojdasiewicz LA, Poniatowski D, Szukiewicz D.The role of inflammatory and anti-inflammatory cytokines in the pathogenesis of osteoarthritis. Mediators Inflamm. 2014;2014:561459–561419. doi:10.1155/2014/561459. 24876674 PMC4021678

[43] Chauhan K. Rheumatoid arthritis-StatPearls-NCBI bookshelf. Treasure Island (FL): StatPearls; 2020.

[44] Cojocaru M, Cojocaru IM, Silosi I, et al. Extra-articular manifestations in rheumatoid arthritis. Maedica (Bucur). 2010;5(4):286–291. 21977172 PMC3152850

[45] Guo Q, Wang Y, Xu D, et al. Rheumatoid arthritis: pathological mechanisms and modern pharmacologic therapies. Bone Res. 2018;6(1):15. doi:10.1038/s41413-018-0016-9. 29736302 PMC5920070

[46] Rossides M. Rheumatoid arthritis and COPD: thinking beyond smoking. Chest. 2024;165(6):1278–1279. doi:10.1016/j.chest.2024.03.005. 38852958

[47] Wang M, Pan H, Zhai Y, et al. Bidirectional association between rheumatoid arthritis and chronic obstructive pulmonary disease: a systematic review and meta-analysis. Front Immunol. 2024;15:1494003. doi:10.3389/fimmu.2024.1494003. 39687614 PMC11647564

[48] Jun KR, Choi SE, Cha CH, et al. Meta-analysis of the association between HLA-DRB1 allele and rheumatoid arthritis susceptibility in Asian populations. J Korean Med Sci. 2007;22(6):973–80. doi:10.3346/jkms.2007.22.6.973. PMC2694263 18162709

[49] Klareskog L, Stolt P, Lundberg K, et al. A new model for an etiology of rheumatoid arthritis: smoking may trigger HLA–DR (shared epitope)–restricted immune reactions to autoantigens modified by citrullination. Arthritis Rheum. 2006;54(1):38–46. doi:10.1002/art.21575. 16385494

[50] Cao Z, Li Q, Wu J, et al. Causal association of rheumatoid arthritis with obstructive lung disease: evidence from Mendelian randomization study. Heart Lung. 2023;62:35–42. doi:10.1016/j.hrtlng.2023.05.020. 37302263

[51] Ungprasert P, Srivali N, Cheungpasitporn W, et al. Risk of incident chronic obstructive pulmonary disease in patients with rheumatoid arthritis: a systematic review and meta-analysis. Joint Bone Spine. 2016;83(3):290–294. doi:10.1016/j.jbspin.2015.05.016. 26709254

[52] Friedlander HM, Ford JA, Zaccardelli A, et al. Obstructive lung diseases and risk of rheumatoid arthritis. Expert Rev Clin Immunol. 2020;16(1):37–50. doi:10.1080/1744666X.2019.1698293. 31774329 PMC6980732

[53] Hemminki K, Liu X, Ji J, et al. Subsequent COPD and lung cancer in patients with autoimmune disease. Eur Respir J. 2011;37(2):463–465. doi:10.1183/09031936.00070410. 21282811

[54] Sparks JA, Lin T-C, Camargo CA, Jr, et al. Rheumatoid arthritis and risk of chronic obstructive pulmonary disease or asthma among women: a marginal structural model analysis in the Nurses’ Health Study. Semin Arthritis Rheum. 2018;47(5):639–648. doi:10.1016/j.semarthrit.2017.09.005. 29037522 PMC5857435

[55] Nannini C, Medina-Velasquez YF, Achenbach SJ, et al. Incidence and mortality of obstructive lung disease in rheumatoid arthritis: a population-based study. Arthritis Care Res (Hoboken). 2013;65(8):1243–1250. doi:10.1002/acr.21986. 23436637 PMC4017238

[56] Dougados M, Soubrier M, Antunez A, et al. Prevalence of comorbidities in rheumatoid arthritis and evaluation of their monitoring: results of an international, cross-sectional study (COMORA). Ann Rheum Dis. 2014;73(1):62–68. doi:10.1136/annrheumdis-2013-204223. 24095940 PMC3888623

[57] Bieber V, Cohen AD, Freud T, et al. Autoimmune smoke and fire—coexisting rheumatoid arthritis and chronic obstructive pulmonary disease: a cross-sectional analysis. Immunol Res. 2013;56(2-3):261–266. doi:10.1007/s12026-013-8395-x. 23568054

[58] Shen T-C, Lin C-L, Chen C-H, et al. Increased risk of chronic obstructive pulmonary disease in patients with rheumatoid arthritis: a population-based cohort study. QJM. 2014;107(7):537–543. doi:10.1093/qjmed/hcu027. 24497528

[59] Ursum J, Nielen MM, Twisk JW, et al. Increased risk for chronic comorbid disorders in patients with inflammatory arthritis: a population based study. BMC Fam Pract. 2013;14(1):199. doi:10.1186/1471-2296-14-199. 24364915 PMC3909051

[60] Mcguire K, Aviña-Zubieta JA, Esdaile JM, et al. Risk of incident chronic obstructive pulmonary disease in rheumatoid arthritis: a population-based cohort study. Arthritis Care Res (Hoboken). 2019;71(5):602–610. doi:10.1002/acr.23410. 29047218

[61] Ameer MA, Chaudhry H, Mushtaq J, et al. An overview of systemic lupus erythematosus (SLE) pathogenesis, classification, and management. Cureus. 2022;14(10):e30330. doi:10.7759/cureus.30330. 36407159 PMC9662848

[62] Cojocaru M, Cojocaru IM, Silosi I, et al. Manifestations of systemic lupus erythematosus. Maedica (Bucur). 2011;6(4):330–336. 22879850 PMC3391953

[63] McVeigh ED, Batool A, Stromberg A, et al. Cardiovascular complications of systemic lupus erythematosus: impact of risk factors and therapeutic efficacy – a tertiary centre experience in an Appalachian state. Lupus Sci Med. 2021;8(1):e000467. doi:10.1136/lupus-2020-000467. 33952624 PMC8103370

[64] Shin JI, Lee KH, Park S, et al. Systemic lupus erythematosus and lung involvement: a comprehensive review. J Clin Med. 2022;11(22):6714. doi:10.3390/jcm11226714. 36431192 PMC9698564

[65] Depascale R, Del Frate G, Gasparotto M, et al. Diagnosis and management of lung involvement in systemic lupus erythematosus and Sjögren’s syndrome: a literature review. Ther Adv Musculoskelet Dis. 2021;13:1759720X211040696. doi:10.1177/1759720X211040696. PMC8488521 34616495

[66] Shen T-C, Lin C-L, Chen C-H, et al. Increased risk of chronic obstructive pulmonary disease in patients with systemic lupus erythematosus: a population-based cohort study. PLoS One. 2014;9(3):e91821. doi:10.1371/journal.pone.0091821. 24622340 PMC3951498

[67] Avina-Zubieta A, Sadatsafavi M, Sayre EC, et al. Elevated risk of chronic obstructive pulmonary disease in systemic lupus erythematosus: a population-based study. Arthritis Res Ther. 2014;16(S1):1–23. doi:10.1186/ar4650.

[68] Katz P, Pedro S, Trupin L. The impact of asthma and Chronic Obstructive Pulmonary Disease (COPD) on patient. ACR Open Rheumatol. 2021;3(4):221–230. 33609085 10.1002/acr2.11212 PMC8063140

[69] Doyle TJ, Dellaripa PF. Lung manifestations in the rheumatic diseases. Chest. 2017;152(6):1283–1295. doi:10.1016/j.chest.2017.05.015. 28552544 PMC5812749

[70] Han G-M, Han X-F. Comorbid conditions are associated with emergency department visits, hospitalizations, and medical charges of patients with systemic lupus erythematosus. J Clin Rheumatol. 2017;23(1):19–25. doi:10.1097/RHU.0000000000000437. 28002152

[71] Reshetnyak T, Nurbaeva K. The Role of Neutrophil Extracellular Traps (NETs) in the pathogenesis of systemic lupus erythematosus and antiphospholipid syndrome. Int J Mol Sci. 2023;24(17):13581. doi:10.3390/ijms241713581. 37686381 PMC10487763

[72] Yu X, Cheng X, Lv L, et al. The association between chronic obstructive pulmonary disease and autoimmune diseases: a bidirectional Mendelian randomization study. Front Med. 2024;11:1331111. doi:10.3389/fmed.2024.1331111. PMC10949139 38504914

[73] Wang X, Wen B, Duan X, et al. Recent advances of type I interferon on the regulation of immune cells and the treatment of systemic lupus erythematosus. J Inflamm Res. 2025;18:4533–4549. doi:10.2147/JIR.S516195. 40182060 PMC11967359

[74] Gumkowska-Sroka O, Kotyla K, Kotyla P. Immunogenetics of systemic sclerosis. Genes (Basel). 2024;15(5):586. doi:10.3390/genes15050586. 38790215 PMC11121022

[75] Liakouli V, Ciancio A, Del Galdo F, et al. Systemic sclerosis interstitial lung disease: unmet needs and potential solutions. Nat Rev Rheumatol. 2024;20(1):21–32. doi:10.1038/s41584-023-01044-x. 37923862

[76] Hassoun PM. Lung involvement in systemic sclerosis. Presse Med. 2011;40(1):3–17. 21195581 10.1016/j.lpm.2010.08.006 PMC3066150

[77] Solomon JJ, Olson AL, Fischer A, et al. Scleroderma lung disease. Eur Respir Rev. 2013;22(127):6–19. doi:10.1183/09059180.00005512. 23457159 PMC4103193

[78] Piplani S, Jelic V, Bejugam VR, et al. COPD outcomes in individuals with scleroderma compared to general population: a population based study. Ann Rheum Dis. 2024;83:1935–1936. doi:10.1136/annrheumdis-2024-eular.2075.

[79] Vakhshoorzadeh J, Lui JK, Sangani R, et al. Chronic obstructive pulmonary disease is associated with increased mortality in systemic sclerosis-related pulmonary hypertension. Am J Respir Crit Care Med. 2022;205:A3029–A3029. doi:10.1164/ajrccm-conference.2022.205.1_MeetingAbstracts.A3029.

[80] Yamakawa H, Takemura T, Iwasawa T, et al. Emphysematous change with scleroderma-associated interstitial lung disease: The potential contribution of vasculopathy? BMC Pulm Med. 2018;18(1):25. doi:10.1186/s12890-018-0591-y. 29382307 PMC5791248

[81] Umit D, Engin C, Kiralp MZ, et al. The pulmonary involvement in rheumatic diseases: pulmonary effects of ankylosing spondylitis and its impact on functionality and quality of life. Tohoku J Exp Med. 2007;212(4):423–430. 17660708 10.1620/tjem.212.423

[82] Sharif K, Watad A, Tiosano S, et al. The link between COPD and ankylosing spondylitis: a population based study. Eur J Intern Med. 2018;53:62–65. doi:10.1016/j.ejim.2018.04.002. 29631757

[83] Fujii U, Miyahara N, Taniguchi A, et al. IL-23 is essential to the development of elastase-induced pulmonary inflammation and emphysema. Airway Cell Biol Immunopathol. 2016:48:60. doi:10.1183/13993003.congress-2016.PA1831. 27351934

[84] Zamout P, Exarchou S, Sharma A, et al. The prevalence of chronic obstructive pulmonary disease in patients with spondyloarthritis compared to the general population in the southernmost region of Sweden: a case–control study. Clin Exp Med. 2024;24(1):75. no doi:10.1007/s10238-024-01335-x. 38598034 PMC11006728

[85] Negrini S, Emmi G, Greco M, et al. Sjögren’s syndrome: a systemic autoimmune disease. Clin Exp Med. 2022;22(1):9–25. doi:10.1007/s10238-021-00728-6. 34100160 PMC8863725

[86] Stefanski A-L, Tomiak C, Pleyer U, et al. The diagnosis and treatment of Sjögren’s syndrome. Deutsches Aerzteblatt Online. 2017;114(20):354–361. 10.3238/arztebl.2017.0354 PMC5471601 28610655

[87] Bálint G, Watson Buchanan W, Kean CA, et al. Sjögren’s syndrome. Inflammopharmacol. 2023;32:37–43. 10.1007/s10787-023-01222-z 37195497

[88] Beydon M, McCoy S, Nguyen Y, et al. Epidemiology of Sjögren syndrome. Nat Rev Rheumatol. 2024;20(3):158–169. doi:10.1038/s41584-023-01057-6. 38110617

[89] Verstappen GM, Pringle S, Bootsma H, et al. Epithelial–immune cell interplay in primary Sjögren syndrome salivary gland pathogenesis. Nat Rev Rheumatol. 2021;17(6):333–348. doi:10.1038/s41584-021-00605-2. 33911236 PMC8081003

[90] Manfrè V, Chatzis L, Cafaro G, et al. Sjögren’s syndrome: one year in review 2022. Clin Exp Rheumatol. 2022;40(12):2211–2224. doi:10.55563/clinexprheumatol/43z8gu. 36541236

[91] Chung A, Wilgus ML, Fishbein G, et al. Pulmonary and bronchiolar involvement in Sjogren’s Syndrome. Semin Respir Crit Care Med. 2019;40(2):235–254. doi:10.1055/s-0039-1688448. 31137063

[92] Berardicurti O, Marino A, Genovali I, et al. Interstitial lung disease and pulmonary damage in primary Sjögren’s syndrome: a systematic review and meta-analysis. J Clin Med. 2023;12(7):2586. doi:10.3390/jcm12072586. 37048669 PMC10095380

[93] Strevens Bolmgren V, Olsson P, Wollmer P, et al. Respiratory symptoms are poor predictors of concomitant chronic obstructive pulmonary disease in patients with primary Sjögren’s syndrome. Rheumatol Int. 2017;37(5):813–818. doi:10.1007/s00296-017-3678-5. 28243798 PMC5397441

[94] Shen T-C, Wu B-R, Chen H-J, et al. Risk of chronic obstructive pulmonary disease in female adults with primary Sjögren Syndrome. Medicine. 2016;95(10):e3066. doi:10.1097/MD.0000000000003066. 26962839 PMC4998920

[95] Nilsson A, Diaz S, Theander E, et al. Chronic obstructive pulmonary disease is common in never-smoking patients with primary Sjögren syndrome. J Rheumatol. 2015;42(3):464–471. doi:10.3899/jrheum.140370. 25593235

[96] Mandl T, Diaz S, Ekberg O, et al. Frequent development of chronic obstructive pulmonary disease in primary SS–results of a longitudinal follow-up. Rheumatology. 2012;51(5):941–946. doi:10.1093/rheumatology/ker409. 22258389

[97] Zhang MZ, Zhang RY, Liu J, et al. Advances in the role of autoimmune mechanisms in chronic obstructive pulmonary disease. Zhonghua Jie He He Hu Xi Za Zhi. 2023;46(11):1131–1136. doi:10.3760/cma.j.cn112147-20230731-00037. 37914427

[98] Mariette X, Criswell LA. Primary Sjögren’s syndrome. N Engl J Med. 2018;378(10):931–939. doi:10.1056/NEJMcp1702514. 29514034

[99] Brightling C, Greenin N. Airway inflammation in COPD: progress to precision medicine. Eur Respir J. 2019;54(2):1900651. doi:10.1183/13993003.00651-2019. 31073084

[100] Hernández-Molina G, Leal-Alegre G, Michel-Peregrina M. The meaning of anti-Ro and anti-La antibodies in primary Sjögren’s syndrome. Autoimmun Rev. 2011;10(3):123–125. doi:10.1016/j.autrev.2010.09.001. 20833272

[101] Wen L, Krauss-Etschmann S, Petersen F, et al. Autoantibodies in chronic obstructive pulmonary disease. Front Immunol. 2018;9:66. doi:10.3389/fimmu.2018.00066. 29422903 PMC5788885

[102] Ramos-Casals M, Brito-Zerón P, Bombardieri S, et al. EULAR recommendations for the management of Sjögren’s syndrome with topical and systemic therapies. Ann Rheum Dis. 2020;79(1):3–18. doi:10.1136/annrheumdis-2019-216114. 31672775

[103] Raharja A, Mahil SK, Barker JN. Psoriasis: a brief overview. Clin Med. 2021;21(3):170–173. doi:10.7861/clinmed.2021-0257. PMC8140694 34001566

[104] Harden JL, Krueger JG, Bowcock AM. The immunogenetics of Psoriasis: a comprehensive review. J Autoimmun. 2015;64:66–73. doi:10.1016/j.jaut.2015.07.008. 26215033 PMC4628849

[105] Lowes MA, Suárez-Fariñas M, Krueger JG. Immunology of psoriasis. Annu Rev Immunol. 2014;32(1):227–255. doi:10.1146/annurev-immunol-032713-120225. 24655295 PMC4229247

[106] Lembke S, Macfarlane GJ, Jones GT. The worldwide prevalence of psoriatic arthritis – a systematic review and meta-analysis. Rheumatology. 2024;2024:keae198. 10.1093/rheumatology/keae198 PMC11637478 38530786

[107] Azuaga AB, Ramírez J, Cañete JD. Psoriatic arthritis: pathogenesis and targeted therapies. Int J Mol Sci. 2023;24(5):4901. doi:10.3390/ijms24054901. 36902329 PMC10003101

[108] Robinson D, Hackett MV, Wong JB, et al. Co-occurrence and comorbidities in patients with immune-mediated inflammatory disorders: an exploration using US healthcare claims data, 2001–2002. Curr Med Res Opin. 2006;22(5):989–1000. doi:10.1185/030079906X104641. 16709321

[109] Matusiewicz A, Stróżyńska-Byrska J, Olesińska M. Polyautoimmunity in rheumatological conditions. Int J Rheum Dis. 2019;22(3):386–391. doi:10.1111/1756-185X.13454. 30548416

[110] Liu Y, Krueger JG, Bowcock AM. Psoriasis: genetic associations and immune system changes. Genes Immun. 2007;8(1):1–12. doi:10.1038/sj.gene.6364351. 17093502

[111] Li X, Kong L, Li F, et al. Association between psoriasis and chronic obstructive pulmonary disease: a systematic review and meta-analysis. PLoS One. 2015;10(12):e0145221. doi:10.1371/journal.pone.0145221. 26700640 PMC4689442

[112] Liu M, Zhou X, Zhang G, et al. The causal relationship between psoriasis and chronic obstructive pulmonary disease: a bidirectional mendelian randomization study. Skin Res Technol. 2024;30(3):629. doi:10.1111/srt.13629. PMC10895548 38407525

[113] Khraishi M, MacDonald D, Rampakakis E, et al. Prevalence of patient-reported comorbidities in early and established psoriatic arthritis cohorts. Clin Rheumatol. 2011;30(7):877–885. doi:10.1007/s10067-011-1692-7. 21287359

[114] Li H, Zuo J, Tang W. Phosphodiesterase-4 Inhibitors for the treatment of inflammatory diseases. Front Pharmacol. 2018;9:1048. doi:10.3389/fphar.2018.01048. 30386231 PMC6199465

[115] Li G, He D, Cai X, et al. Advances in the development of phosphodiesterase-4 inhibitors. Eur J Med Chem. 2023;250:115195. doi:10.1016/j.ejmech.2023.115195. 36809706

[116] Quartuccio L, Sebastiani M, Spinelli FR, et al. More than a random association between chronic obstructive pulmonary disease and psoriatic arthritis: shared pathogenic features and implications for treatment. Expert Rev Clin Immunol. 2022;18(9):983–990. doi:10.1080/1744666X.2022.2106969. 35881045

[117] Knight JS, Branch DW, Ortel TL. Antiphospholipid syndrome: advances in diagnosis, pathogenesis, and management. BMJ. 2023;380:e069717. doi:10.1136/bmj-2021-069717. 36849186

[118] Gaspar P, Cohen H, Isenberg DA. The assessment of patients with the antiphospholipid antibody syndrome: Where are we now? Rheumatology. 2020;59(7):1489–1494. doi:10.1093/rheumatology/keaa172. 32359070

[119] Xourgia E, Tektonidou MG. An update on antiphospholipid syndrome. Curr Rheumatol Rep. 2022;23(12):84. no doi:10.1007/s11926-021-01051-5. 34985625

[120] Tohidi-Esfahani I, Mittal P, Isenberg D, et al. Platelets and thrombotic antiphospholipid syndrome. J Clin Med. 2024;13(3):741. doi:10.3390/jcm13030741. 38337435 PMC10856779

[121] Carmi O, Berla M, Shoenfeld Y, et al. Diagnosis and management of catastrophic antiphospholipid syndrome. Expert Rev Hematol. 2017;10(4):365–374. doi:10.1080/17474086.2017.1300522. 28277850

[122] Maioli G, Calabrese G, Capsoni F, et al. Lung disease in antiphospholipid syndrome. Semin Respir Crit Care Med. 2019;40(2):278–294. doi:10.1055/s-0039-1683994. 31137066

[123] Ford HJ, Roubey RAS. Pulmonary manifestations of the antiphospholipid antibody syndrome. Clin Chest Med. 2010;31(3):537–545. doi:10.1016/j.ccm.2010.05.005. 20692545

[124] Nesher G, Breuer GS. Giant cell arteritis and polymyalgia rheumatica: 2016 update. Rambam Maimonides Med J. 2016;7(4):2016. doi:10.5041/RMMJ.10262. PMC5101009 27824543

[125] Nesher G. Polymyalgia rheumatica – diagnosis and classification. J Autoimmun. 2014;48-49:76–78. doi:10.1016/j.jaut.2014.01.016. 24461540

[126] Dasgupta B, Cimmino MA, Maradit-Kremers H, et al. 2012 provisional classification criteria for polymyalgia rheumatica: a European League Against Rheumatism/American College of Rheumatology collaborative initiative. Ann Rheum Dis. 2012;71(4):484–492. doi:10.1136/annrheumdis-2011-200329. 22388996 PMC3298664

[127] Floris A, Piga M, Cauli A, et al. Polymyalgia rheumatica: an autoinflammatory disorder? RMD Open. 2018;4(1):e000694. doi:10.1136/rmdopen-2018-000694. 29955386 PMC6018871

[128] Uddhammar A, Boman J, Juto P, et al. Antibodies against Chlamydia pneumoniae, cytomegalovirus, enteroviruses and respiratory syncytial virus in patients with polymyalgia rheumatica. Clin Exp Rheumatol. 1997;15(3):299–302. 9177926

[129] Hammoda HMB, Al Saleh J, Mahmood K, et al. Polymyalgia Rheumatica (PMR) and lung involvement: the forgotten association. Oman Med J. 2020;35(2):e105. doi:10.5001/omj.2020.23. 32181007 PMC7060987

[130] Sambataro G, Sambataro D, Pignataro F, et al. Interstitial lung disease in patients with Polymyalgia Rheumatica: a case series. Respir Med Case Rep. 2019;26:126–130. doi:10.1016/j.rmcr.2018.12.014. 30603602 PMC6307098

[131] Cocquempot K, Defuentes G, Duron-Martineau S, et al. Polymyalgia rheumatica revealing a lung cancer. Rev Mal Respir. 2013;30(1):67–70. doi:10.1016/j.rmr.2012.06.015. 23318192

[132] Coelho S, Magalhães H, Correia J, et al. Polymyalgia rheumatica and pulmonary adenocarcinoma: a case report and literature review. Porto Biomed J. 2017;2(3):93–95. doi:10.1016/j.pbj.2017.01.007. 32258595 PMC6806980

[133] Baumgart DC, Carding SR. Inflammatory bowel disease: cause and immunobiology. Lancet. 2007;369(9573):1627–−1640. doi:10.1016/S0140-6736(07)60750-8. 17499605

[134] Burisch J, Jess T, Martinato M, et al. The burden of inflammatory bowel disease in Europe. J Crohns Colitis. 2013;7(4):322–337. doi:10.1016/j.crohns.2013.01.010. 23395397

[135] Ng SC, Shi HY, Hamidi N, et al. Worldwide incidence and prevalence of inflammatory bowel disease in the 21st century: a systematic review of population-based studies. Lancet. 2017;390(10114):2769–2778. doi:10.1016/S0140-6736(17)32448-0. 29050646

[136] Zippi M, Corrado C, Pica R, et al. Extraintestinal manifestations in a large series of Italian inflammatory bowel disease patients. World J Gastroenterol. 2014;20(46):17463–17467. doi:10.3748/wjg.v20.i46.17463. 25516659 PMC4265606

[137] Hedin CR, Vavricka SR, Stagg AJ, et al. The pathogenesis of extraintestinal manifestations: implications for IBD research, diagnosis, and therapy. J Crohns Colitis. 2019;13(5):541–554. doi:10.1093/ecco-jcc/jjy191. 30445584

[138] Lakatos L, Pandur T, David G, et al. Association of extraintestinal manifestations of inflammatory bowel disease in a province of western Hungary with disease phenotype: results of a 25-year follow-up study. World J Gastroenterol. 2003;9(10):2300–2307. doi:10.3748/wjg.v9.i10.2300. 14562397 PMC4656482

[139] Bernstein CN, Blanchard JF, Rawsthorne P, et al. The prevalence of extraintestinal diseases in inflammatory bowel disease: a population-based study. Am J Gastroenterol. 2001;96(4):1116–1122. doi:10.1111/j.1572-0241.2001.03756.x. 11316157

[140] Vutcovici M, Brassard P, Bitton A. Inflammatory bowel disease and airway diseases. World J Gastroenterol. 2016;22(34):7735–7741. doi:10.3748/wjg.v22.i34.7735. 27678355 PMC5016372

[141] Kinose D, Ogawa E, Hirota T, et al. A NOD2 gene polymorphism is associated with the prevalence and severity of chronic obstructive pulmonary disease in a Japanese population. Respirology. 2012;17(1):164–171. 21943069 10.1111/j.1440-1843.2011.02069.x

[142] Vargas-Rojas MI, Ramirez-Venegas A, Limon-Camacho L, et al. Increase of Th17 cells in peripheral blood of patients with chronic obstructive pulmonary disease. Respir Med. 2011;105(11):1648–1654. doi:10.1016/j.rmed.2011.05.017. 21763119

[143] Eastaff-Leung N, Mabarrack N, Barbour A, et al. Foxp3+ regulatory T cells, Th17 effector cells, and cytokine environment in inflammatory bowel disease. J Clin Immunol. 2010;30(1):80–89. doi:10.1007/s10875-009-9345-1. 19936899

[144] AL, Raftery E, Tsantikos E, NL, Harris, et al. Links between inflammatory bowel disease and chronic obstructive pulmonary disease. Front Immunol. 2020;11(11):2144. 33042125 10.3389/fimmu.2020.02144 PMC7517908

[145] Keely S, Talley NJ, Hansbro PM. Pulmonary-intestinal cross-talk in mucosal inflammatory disease. Mucosal Immunol. 2012;5(1):7–18. doi:10.1038/mi.2011.55. 22089028 PMC3243663

[146] Zergham AS, Sekhon AK, Mebasher A, et al. Inflammatory bowel disease and obstructive pulmonary disease: A two-way association? Cureus. 2020;12(1):e6836. doi:10.7759/cureus.6836. 32181078 PMC7051109

[147] Zhao Y, Wang J, Liu Z, et al. Pulmonary dysfunction in 114 patients with inflammatory bowel disease. Medicine. 2017;96(18):e6808. doi:10.1097/MD.0000000000006808. 28471982 PMC5419928

[148] Vutcovici M, Bitton A, Ernst P, et al. Inflammatory bowel disease and risk of mortality in COPD. Eur Respir J. 2016;47(5):1357–1364. doi:10.1183/13993003.01945-2015. 26869671

[149] Gupta SJ, Gupta VL, Kothari HG, et al. Assessment of occult pulmonary involvement in ulcerative colitis. Inflamm Intest Dis. 2020;5(3):144–150. doi:10.1159/000508772. 32999887 PMC7506263

[150] Georgakopoulou VE, Tarantinos K, Papalexis P, et al. Role of pulmonary function testing in inflammatory bowel diseases. Med Int. 2022;2(4):25. doi:10.3892/mi.2022.50. PMC9829212 36699508

[151] Jacobsen HA, Karachalia Sandri A, Weinreich UM, et al. Increased risk of obstructive lung disease in inflammatory bowel disease: a population-based cohort study. United European Gastroenteroly J. 2024;12(4):477–486. doi:10.1002/ueg2.12527. PMC11091783 38183388

[152] Valentin S, Renel B, Manneville F, et al. Prevalence of and factors associated with respiratory symptoms among patients with inflammatory bowel disease: a prospective study. Inflamm Bowel Dis. 2023;29(2):207–216. doi:10.1093/ibd/izac062. 35394504

[153] Pemmasani G, Loftus EV, Tremaine WJ. Prevalence of pulmonary diseases in association with inflammatory Bowel disease. Dig Dis Sci. 2022;67(11):5187–5194. doi:10.1007/s10620-022-07385-z. 35142913

[154] Lee J, Im JP, Han K, et al. Risk of inflammatory bowel disease in patients with chronic obstructive pulmonary disease: a nationwide, population-based study. World J Gastroenterol. 2019;25(42):6354–6364. doi:10.3748/wjg.v25.i42.6354. 31754295 PMC6861849

[155] Brassard P, Vutcovici M, Ernst P, et al. Increased incidence of inflammatory bowel disease in Québec residents with airway diseases. Eur Respir J. 2015;45(4):962–968. doi:10.1183/09031936.00079414. 25406447

[156] Ekbom A, Brandt L, Granath F, et al. Increased risk of both ulcerative colitis and Crohn’s disease in a population suffering from COPD. Lung. 2008;186(3):167–172. doi:10.1007/s00408-008-9080-z. 18330638

[157] Ludvigsson JF, Inghammar M, Ekberg M, et al. A nationwide cohort study of the risk of chronic obstructive pulmonary disease in coeliac disease. J Intern Med. 2012;271(5):481–489. doi:10.1111/j.1365-2796.2011.02448.x. 21880073

[158] Ordás I, Eckmann L, Talamini M, et al. Ulcerative colitis. Lancet. 2012;380(9853):1606–1619. doi:10.1016/S0140-6736(12)60150-0. 22914296

[159] Catassi C, Verdu EF, Bai JC, et al. Coeliac disease. Lancet. 2022;399(10344):2413–2426. doi:10.1016/S0140-6736(22)00794-2. 35691302

[160] Cavassani A, Berté ÁD, Pinto AR, et al. Definition and characteristics of multiple sclerosis with predominant cognitive presentation. J Exp Neurol. 2024;5(1):40–41. doi:10.33696/Neurol.5.086.

[161] McBenedict B, Goh KS, Yau RCC, et al. Neuropathic pain secondary to multiple sclerosis: a narrative review. Cureus. 2024;16(6):e61587. doi:10.7759/cureus.61587. 38962595 PMC11221503

[162] Ashtari F, Esmaeil N, Mansourian M, et al. An 8-year study of people with multiple sclerosis in Isfahan, Iran: association between environmental air pollutants and severity of disease. J Neuroimmunol. 2018;319:106–111. doi:10.1016/j.jneuroim.2018.02.019. 29526408

[163] Marrie RA, Patten S, Tremlett H, et al. Chronic lung disease and multiple sclerosis: incidence, prevalence, and temporal trends. Mult Scler Relat Disord. 2016;8:86–92. doi:10.1016/j.msard.2016.05.009. 27456880

[164] Kang J-H, Chen Y-H, Lin H-C. Comorbidities amongst patients with multiple sclerosis: a population-based controlled study. Eur J Neurol. 2010;17(9):1215–1219. doi:10.1111/j.1468-1331.2010.02971.x. 20192982

[165] Lei Z, Lin W. Mechanisms governing oligodendrocyte viability in multiple sclerosis and its animal models. Cells. 2024;13(2):116. doi:10.3390/cells13020116. 38247808 PMC10814231

[166] Goodin DS. The genetic basis of multiple sclerosis: a model for MS susceptibility. BMC Neurol. 2010;10:101. no doi:10.1186/1471-2377-10-101. 21029420 PMC2994805

[167] Ghoshouni H, Rafiei N, Panah MY, et al. Asthma and chronic obstructive pulmonary disease (COPD) in people with multiple sclerosis: a systematic review and meta-analysis. Mult Scler Relat Disord. 2024;85:105546. doi:10.1016/j.msard.2024.105546. 38507873

[168] Burke H, Wilkinson TMA. Unravelling the mechanisms driving multimorbidity in COPD to develop holistic approaches to patient-centred care. Eur Respir Rev. 2021;30(160):210041. doi:10.1183/16000617.0041-2021. 34415848 PMC9488970

[169] Hlapčić I, Belamarić D, Bosnar M, et al. Combination of systemic inflammatory biomarkers in assessment of chronic obstructive pulmonary disease: diagnostic performance and identification of networks and clusters. Diagnostics. 2020;10(12):1029. doi:10.3390/diagnostics10121029. 33266187 PMC7760570

[170] Pantazopoulos I, Magounaki K, Kotsiou O, et al. Incorporating biomarkers in COPD management: the research keeps going. J Pers Med. 2022;12(3):379. doi:10.3390/jpm12030379. 35330379 PMC8955907

[171] Sin DD, Man SFP. Why are patients with chronic obstructive pulmonary disease at increased risk of cardiovascular diseases? The potential role of systemic inflammation in chronic obstructive pulmonary disease. Circulation. 2003;107(11):1514–1519. doi:10.1161/01.cir.0000056767.69054.b3. 12654609

[172] Agustí AGN, Noguera A, Sauleda J, et al. Systemic effects of chronic obstructive pulmonary disease. Eur Respir J. 2003;21(2):347–360. doi:10.1183/09031936.03.00405703. 12608452

[173] Faner R, Cruz T, Agusti A. Immune response in chronic obstructive pulmonary disease. Expert Rev Clin Immunol. 2013;9(9):821–833. doi:10.1586/1744666X.2013.828875. 24070046

[174] Kany S, Vollrath JT, Relja B. Cytokines in Inflammatory Disease. Int J Mol Sci. 2019;20(23):6008. doi:10.3390/ijms20236008. 31795299 PMC6929211

[175] Moser T, Akgün K, Proschmann U, et al. The role of TH17 cells in multiple sclerosis: therapeutic implications. Autoimmun Rev. 2020;19(10):102647. doi:10.1016/j.autrev.2020.102647. 32801039

[176] Peeters LM, Vanheusden M, Somers V, et al. Cytotoxic CD4+ T cells drive multiple sclerosis progression. Front Immunol. 2017;8:1160. doi:10.3389/fimmu.2017.01160. 28979263 PMC5611397

[177] Purohit M, Gupta G, Afzal O, et al. Janus kinase/signal transducers and activator of transcription (JAK/STAT) and its role in Lung inflammatory disease. Chem Biol Interact. 2023;371:110334. doi:10.1016/j.cbi.2023.110334. 36610610

[178] Calderon AA, Dimond C, Choy DF, et al. Targeting interleukin-33 and thymic stromal lymphopoietin pathways for novel pulmonary therapeutics in asthma and COPD. Eur Respir Rev. 2023;32(167):220144. doi:10.1183/16000617.0144-2022. 36697211 PMC9879340

[179] Burakgazi AZ, Höke A. Respiratory muscle weakness in peripheral neuropathies. J Peripher Nerv Syst. 2010;15(4):307–313. doi:10.1111/j.1529-8027.2010.00293.x. 21199102

[180] Khanna M, Rawat N, Gupta A, et al. Pulmonary involvement in patients with Guillain–Barré Syndrome in subacute phase. J Neurosci Rural Pract. 2017;8(3):412–416. doi:10.4103/jnrp.jnrp_11_17. 28694622 PMC5488563

[181] Wijdicks EFM, Klein CJ. Guillain-Barré Syndrome. Mayo Clin Proc. 2017;92(3):467–479. doi:10.1016/j.mayocp.2016.12.002. 28259232

[182] van den Berg B, Walgaard C, Drenthen J, et al. Guillain–Barré syndrome: pathogenesis, diagnosis, Treatment and Prognosis. Nat Rev Neurol. 2014;10(8):469–482. doi:10.1038/nrneurol.2014.121. 25023340

[183] Mohammadi S, Moosaie F, Aarabi MH. Understanding the immunologic characteristics of neurologic manifestations of SARS-CoV-2 and potential immunological mechanisms. Mol Neurobiol. 2020;57(12):5263–5275. doi:10.1007/s12035-020-02094-y. 32869183 PMC7458880

[184] Israeli E, Agmon-Levin N, Blank M, et al. Guillain–Barré syndrome – A classical autoimmune disease triggered by infection or vaccination. Clin Rev Allergy Immunol. 2012;42(2):121–130. doi:10.1007/s12016-010-8213-3. 20890797

[185] Vahabi M, Ghazanfari T, Sepehrnia S. Molecular mimicry, hyperactive immune system, and SARS-COV-2 are three prerequisites of the autoimmune disease triangle following COVID-19 infection. Int Immunopharmacol. 2022;112:109183. doi:10.1016/j.intimp.2022.109183. 36182877 PMC9393178

[186] Davidson I, Parker ZJ. Falls in people post-Guillain-Barré syndrome in the United Kingdom: a national cross-sectional survey of community based adults. Health Social Care Comm. 2022;30(5):2590–2603. doi:10.1111/hsc.13703. PMC9546005 35015326

[187] Aggarwal AN, Gupta D, Lal V, et al. Ventilatory management of respiratory failure in patients with severe Guillain-Barre syndrome. Neurol India. 2003;51(2):203–205. 14571003

[188] Mozhdehipanah H, Paybast S, Gorji R. Guillain–Barré syndrome as a neurological complication of COVID-19 infection: a case series and review of the literature. Int Clin Neurosci J. 2020;7(3):156–161. doi:10.34172/icnj.2020.18.

[189] García-Manzanedo S, López de la Oliva Calvo L, Ruiz Álvarez L. Guillain-Barré syndrome after Covid-19 infection. Med Clin. 2020;155(8):366. doi:10.1016/j.medcle.2020.06.019. PMC7526591 33020737

[190] Ammouri W, Harmouche H, Khibri H, et al. Pernicious Anaemia: mechanisms, diagnosis, and management. EMJ Hematol. 2020;1(1):71–80. doi:10.33590/emjhematolus/19-00187.

[191] Batool S, Iqbal R. Macrocytic anemia: a review. J Entomol Zool Stud. 2016;4(5):544–547.

[192] Takahashi N, Kameoka J, Takahashi N, et al. Causes of macrocytic anemia among 628 patients: mean corpuscular volumes of 114 and 130 fL as critical markers for categorization. Int J Hematol. 2016;104(3):344–357. doi:10.1007/s12185-016-2043-x. 27352093

[193] Wacka E, Nicikowski J, Jarmuzek P, et al. Anemia and its connections to inflammation in older adults: a review. J Clin Med. 2024;13(7):2049. doi:10.3390/jcm13072049. 38610814 PMC11012269

[194] Lee Y-G, Chang Y, Kang J, et al. Risk factors for incident anemia of chronic diseases: A cohort study. PLoS One. 2019;14(5):e0216062. doi:10.1371/journal.pone.0216062. 31059543 PMC6502324

[195] Kovchun AV, Smiianov VA, Kuchma NG, et al. The impact of systemic inflammation on anemia in patients with chronic obstructive pulmonary disease. Wiad Lek. 2020;73(2):325–328. doi:10.36740/WLek202002123. 32248169

[196] Tsantes AE, Papadhimitriou SI, Tassiopoulos ST, et al. Red cell macrocytosis in hypoxemic patients with chronic obstructive pulmonary disease. Respir Med. 2004;98(11):1117–1123. doi:10.1016/j.rmed.2004.04.002. 15526813

[197] Gadó K, Khodier M, Virág A, et al. Anemia of geriatric patients. Physiol Int. 2022;109(2):119–134. doi:10.1556/2060.2022.00218. 35895570

[198] Almario CV, Metz DC, Haynes K, et al. Risk of community-acquired pneumonia in patients with a diagnosis of pernicious anemia. Europ J Gastroenterol Hepatol. 2015;27(11):1259–1264. doi:10.1097/MEG.0000000000000444. PMC4586398 26225868

[199] Szablewski L. Role of immune system in type 1 diabetes mellitus pathogenesis. Int Immunopharmacol. 2014;22(1):182–191. doi:10.1016/j.intimp.2014.06.033. 24993340

[200] Mukhtar Y, Galalain A, Yunusa U. A modern overview on diabetes mellitus: a chronic endocrine disorder. EJB. 2020;5(2):1–14. doi:10.47672/ejb.409.

[201] Kahanovitz L, Sluss PM, Russell SJ. Type 1 diabetes – a clinical perspective. Point Care. 2017;16(1):37–40. doi:10.1097/POC.0000000000000125. 28943810 PMC5606981

[202] Crisafulli E, Guerrero M, Menéndez R, et al. Inhaled corticosteroids do not influence the early inflammatory response and clinical presentation of hospitalized subjects with COPD exacerbation. Respir Care. 2014;59(10):1550–1559. doi:10.4187/respcare.03036. 25074943

[203] Ehrlich SF, Quesenberry CP, Eeden SKVD, et al. Patients diagnosed with diabetes are at increased risk for asthma, chronic obstructive pulmonary disease, pulmonary fibrosis, and pneumonia but not lung cancer. Diabetes Care. 2010;33(1):55–60. doi:10.2337/dc09-0880. 19808918 PMC2797986

[204] O’Byrne PM, Rennard S, Gerstein H, et al. Risk of new onset diabetes mellitus in patients with asthma or COPD taking inhaled corticosteroids. Respir Med. 2012;106(11):1487–1493. 22902134 10.1016/j.rmed.2012.07.011

[205] Caughey GE, Preiss AK, Vitry AI, et al. Comorbid Diabetes and COPD: impact of corticosteroid use on diabetes complications. Diabetes Care. 2013;36(10):3009–3014. doi:10.2337/dc12-2197. 23735725 PMC3781532

[206] Ho T-W, Huang C-T, Ruan S-Y, et al. Diabetes mellitus in patients with chronic obstructive pulmonary disease-The impact on mortality. PLoS One. 2017;12(4):e0175794. doi:10.1371/journal.pone.0175794. 28410410 PMC5391945

[207] Visca D, Pignatti P, Spanevello A, et al. Relationship between diabetes and respiratory diseases – clinical and therapeutic aspects. Pharmacol Res. 2018;137:230–235. doi:10.1016/j.phrs.2018.10.008. 30312663

[208] Park SS, Perez Perez JL, Perez Gandara B, et al. Mechanisms linking COPD to type 1 and 2 diabetes mellitus: Is there a relationship between diabetes and COPD? Medicina (B Aires). 2022;58(8):1030. doi:10.3390/medicina58081030. PMC9415273 36013497

[209] Vlahos R, Bozinovski S. Role of alveolar macrophages in chronic obstructive pulmonary disease. Front Immunol. 2014;5:435. no doi:10.3389/fimmu.2014.00435. 25309536 PMC4160089

[210] Mercado N, Ito K, Barnes PJ. Accelerated ageing of the lung in COPD: new concepts. Thorax. 2015;70(5):482–489. doi:10.1136/thoraxjnl-2014-206084. 25739910

[211] Donath MY, Shoelson SE. Type 2 diabetes as an inflammatory disease. Nat Rev Immunol. 2011;11(2):98–107. doi:10.1038/nri2925. 21233852

[212] Ghowsi M, Qalekhani F, Farzaei MH, et al. Inflammation, oxidative stress, insulin resistance, and hypertension as mediators for adverse effects of obesity on the brain: a review. Biomedicine. 2021;11(4):13–22. doi:10.37796/2211-8039.1174. 35223415 PMC8823488

[213] Janciauskiene S, Welte T. Well-known and less well-known functions of Alpha-1 antitrypsin. Its role in chronic obstructive pulmonary disease and other disease developments. Ann Am Thorac Soc. 2016;13(Suppl 4):S280–S288. doi:10.1513/AnnalsATS.201507-468KV. 27564662

[214] Lewis EC, Mizrahi M, Toledano MB, et al. α1-Antitrypsin monotherapy induces immune tolerance during islet allograft transplantation in mice. National Acad Sci. 2008;105(42):16236–16241. doi:10.1073/pnas.0807627105. PMC2566995 18852465

[215] Rogliani P, Lucà G, Lauro D. Chronic obstructive pulmonary disease and diabetes. COPD Res Pract. 2015;1(1):5. doi:10.1186/s40749-015-0005-y.

[216] Agbor DBA, Kari M, Chukka RCH, et al. Prevalence and impact of thyroid dysfunction in patients with chronic pulmonary obstructive pulmonary disorder: a systematic review and meta-analysis. Cureus. 2024;16(2):e54968. 10.7759/cureus.54968 PMC10972545 38544598

[217] Akpinar EE. An underestimated comorbidity of COPD: thyroid dysfunction. Tuberk Toraks. 2019;67(2):131–135. 31414644 10.5578/tt.68257

[218] Miłkowska-Dymanowska J, Białas AJ, Laskowska P, et al. Thyroid gland in chronic obstructive pulmonary disease. Adv Respir Med. 2017;85(1):28–34. doi:10.5603/ARM.2017.0006. 28198991

[219] Dimopoulou I, Ilias I, Mastorakos G, et al. Effects of severity of chronic obstructive pulmonary disease on thyroid function. Metabolism. 2001;50(12):1397–1401. doi:10.1053/meta.2001.28157. 11735083

[220] Fatih K, Kiraz ZK, Kocatürk E, et al. The level of serum C-reactive protein and neutrophil lymphocyte ratio according to thyroid function status. Clin Experimental Health Sci. 2020;10(2):142–147.

[221] El-Yazed HAA, El-Bassiony MRA, Eldaboosy SAM, et al. Assessment of thyroid functions in patients with chronic obstructive pulmonary disease. Egypt J Chest Dis Tuberc. 2013;62(3):387–391.

[222] Singh L, Jain A, Agrawal A, et al. A study of prevalence of thyroid disorders in chronic obstructive pulmonary disease patients at a tertiary care center in UP. Int J Contemporary Med Res. 2016;3:1239–1242.

[223] Gumus A, Ozcelik N, Yilmaz Kara B, et al. thyroid gland disease as a comorbid condition in COPD. Pulm Med. 2021;2021(1):7479992–7479996. doi:10.1155/2021/7479992. 34745661 PMC8570902

[224] Huang D, Wu D, He J, et al. Association between thyroid function and acute exacerbation of chronic obstructive pulmonary disease. Int J Chron Obstruct Pulmon Dis. 2021;16:333–339. doi:10.2147/COPD.S291807. 33628017 PMC7898213

[225] Sebasan R, Baliga K. Prevalence of thyroid dysfunction in moderate to severe chronic obstructive pulmonary disease patients-a cross-sectional study. Int J Sci Study. 2021;9:160–163.

[226] Terzano C, Romani S, Paone G, et al. COPD and thyroid dysfunctions. Lung. 2014;192(1):103–109. doi:10.1007/s00408-013-9537-6. 24281671

[227] Sarinc Ulasli S, Bozbas SS, Ozen ZE, et al. Effect of thyroid function on COPD exacerbation frequency: a preliminary study. Multidiscip Respir Med. 2013;8(1):64. doi:10.1186/2049-6958-8-64. 24079533 PMC3845712

